# Distinctive metabolic disturbances associated with redox homeostasis, nervous and hormonal functions during gut microbial enrichment upon polystyrene microplastic exposure

**DOI:** 10.1002/imo2.70043

**Published:** 2025-07-23

**Authors:** Guozhu Ye, Zeming Wu, Guoyou Chen, Xuyi Liu, Yifang Duan, Minghui Li, Qiansheng Huang

**Affiliations:** ^1^ Xiamen Key Laboratory of Indoor Air and Health, State Key Laboratory for Ecological Security of Regions and Cities, State Key Laboratory of Advanced Environmental Technology, Institute of Urban Environment Chinese Academy of Sciences Xiamen China; ^2^ iPhenome Biotechnology (Dalian) Inc. Dalian China; ^3^ College of Pharmacy, Daqing Campus Harbin Medical University Daqing China

## Abstract

Microplastic‐induced gut microbial enrichment was dominated by bacteria within Eubacteriales, correlated with the virome, and accompanied by colitis. The polyamine synthetic pathway was activated to maintain glutathionylspermidine homeostasis, concurrent with decreases in pathways involved in the production of energy and reactive oxygen species under microplastic exposure. Tryptophan‐serotonin, phenylalanine‐phenylethylamine, and tyrosine‐thyroxine pathways increased, whereas tryptophan‐kynurenine, tryptophan‐indole, and tyrosine‐tyramine pathways decreased under microplastic exposure. Enterolactone synthesis and cholesterol‐derived hormone synthesis were increased under microplastic exposure. Bacteria within Eubacteriales (e.g., Oscillospiraceae bacterium and Clostridiales bacterium) contributed most to metabolic disturbances under microplastic exposure.

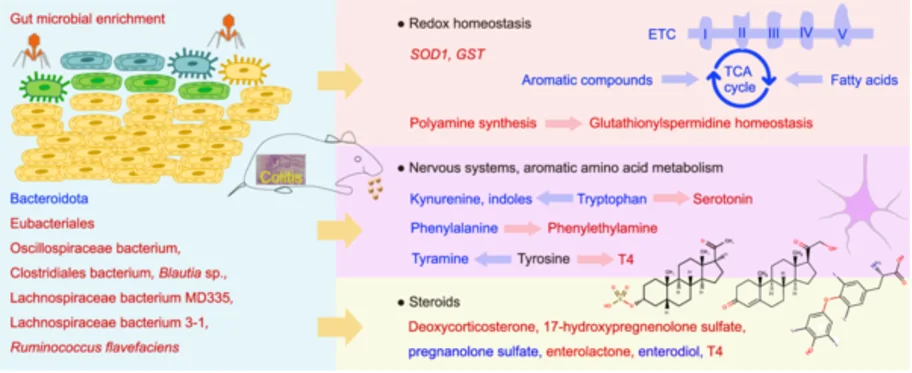

## AUTHOR CONTRIBUTIONS

**Guozhu Ye**: Conceptualization; formal analysis; data curation; funding acquisition; investigation; visualization; Writing—original draft; Writing—review and editing. **Zeming Wu**: Conceptualization; investigation. **Guoyou Chen**: Investigation. **Xuyi Liu**: Investigation. **Yifang Duan**: Investigation. **Minghui Li**: Investigation. **Qiansheng Huang**: Funding acquisition.

## CONFLICT OF INTEREST STATEMENT

The authors declare no conflicts of interest.

## ETHICS STATEMENT

All animal experiments (approval number, HMUDQ20240110011) were approved by the Animal Ethics Committee of Harbin Medical University‐Daqing.

To the Editor,

Microplastics, plastic particles smaller than 5 mm in size, are widely found in the human body, food, drink, and the environment [1, 2]. Studies showed that median contents of microplastics in the brain were 4917 µg/g, 7–10 times higher than those observed in the kidney and liver in decedents, and that higher microplastic accumulations were found in the brain of dementia patients, with notable deposition in cerebrovascular walls [2]. Notably, microplastic levels in the environment and humans still increase [1]. Therefore, the health effects of microplastics, particularly those on the nervous and cerebrovascular systems, warrant particular attention.

Microplastic exposure induces selective enrichment of gut microbiota and resultant dysfunctions [3, 4, 5, 6]. Polystyrene microplastic (PS) exposure increased Bacteroidaceae, Oscillospiraceae, Lachnospiraceae, Deferribacteres, Akkermansiaceae, Marinifilaceae, and *Desulfovibrio*, while decreased Atopobiaceae, *Parabacteroides*, and *Bifidobacterium* in high‐fat diet‐fed mice, concurrent with decreases in the mucin MUC2 and increases in serum lipopolysaccharides and inflammatory factors [4]. However, PS exposure‐induced changes in colonic *Muc2* and *Tjp1*, serum inflammatory factors, and obesity outcomes did not occur in microbiota‐depleted mice [4]. Consistently, colonization of gut microbiota from mice treated with high‐fat diets and PS to microbiota‐depleted mice treated with high‐fat diets led to decreases in mucus proteins, increases in inflammatory factors, and obesity outcomes [4].

Gut microbiota mediates human health and diseases, such as mental disorders [7]. However, molecular mechanisms underlying dysbiosis and dysfunctions of gut microbiota under microplastic exposure are still unclear. Some key unresolved issues include: (1) Selective enrichment characteristics of gut microbiota at the species/strain level and its molecular driving mechanisms; (2) Unique metabolic disorders of gut microbiota and their molecular driving mechanisms; (3) How metabolic factors influence interactions between gut microbiota and the host. We hypothesize that selective gut microbial enrichment and dysfunctions will occur under PS exposure. Therefore, we employed metagenomics to decipher microplastic‐induced selective enrichment characteristics of gut microbiota. Concurrently, unique metabolic disorders in gut microbiota were investigated at the gene level. Subsequently, metabolomics was used to analyze and verify microplastic‐induced distinctive metabolic disorders in gut microbiota, and to identify key metabolic pathways and potential intervention targets.

## 1 RESULTS AND DISCUSSION

**PS exposure induces gut microbial enrichment from the phylum to the species level**

**Figure 1:**
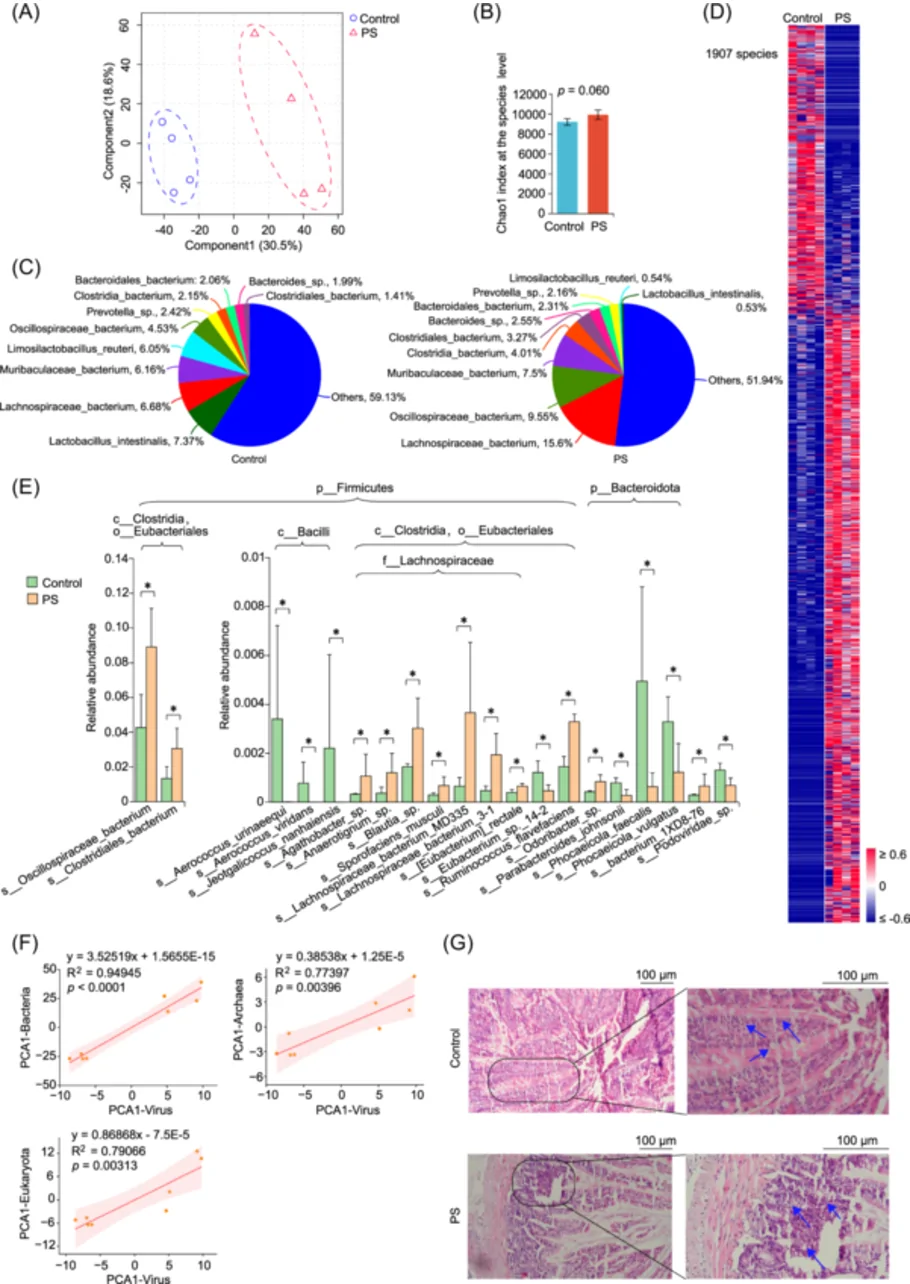
Polystyrene microplastic (PS) exposure induces gut microbial enrichment at the species level. (A) Partial least squares‐discriminant analysis of PS‐induced changes in gut microbiota. (B) PS‐induced changes in the Chao index. (C) PS‐induced changes in the microbial composition. (D) The heat map plot of PS‐induced changes in gut microbiota. Microbes significantly altered (*p* < 0.05, two‐sided Mann–Whitney *U* test) upon PS exposure were all listed. (E) Column plots of PS‐induced changes in gut microbiota. The top 20 abundant microbes with significant alterations upon PS exposure were listed. (F) Correlations of changes in gut virome with those in bacteria, eukaryota, and archaea. (G) Morphological changes in colonic tissues *, *p* < 0.05, two‐sided Mann–Whitney *U* test. The average plus standard deviation was employed for the column plot.

Exposure to 2 µm PS induced changes in the distribution profile of gut microbiota, and increased gut microbiota richness from the phylum to species level (Figures S1−S6, and Figure 1A–C). Notably, 85.29%, 87.27%, 86.67%, 84.24%, 76.84%, and 68.07% of microbes with significant alterations from the phylum to species level were increased, indicating gut microbial enrichment upon PS exposure (Figures S2−S6, and Figure 1D). Among abundant microbes with significant alterations upon PS exposure, 11 bacteria within Eubacteriales, were significantly increased, such as Oscillospiraceae bacterium, Clostridiales bacterium, and Lachnospiraceae bacterium 3–1 (Figure 1E). Conversely, 3 bacteria within Bacilli and 3 of 4 bacteria within Bacteroidota were significantly decreased under PS exposure. These data demonstrated that PS‐induced selective gut microbial enrichment were mainly bacteria within Eubacteriales. Furthermore, changes in bacteria, archaea, and eukaryota were significantly associated with those in viruses, suggesting mediating roles of viruses in gut microbiota under PS exposure (Figure 1F). Concurrently, inflammatory cell infiltration and secretions in glandular ducts, and inflammatory cell infiltration between glandular ducts were observed, indicating colitis in rats exposed to PS (Figure 1G). Exposure to micro/nanoplastics increased the α‐diversity of gut microbiota and shifted the most abundant gut microbes from Lactobacillaceae to Lachnospiraceae, accompanied by hepatocyte swelling, spotty necrosis, karyopyknosis, and cell vacuolization in the liver [8]. Besides, Oscillospiraceae and *Lachnoclostridium* were increased during PS‐induced colonic oxidative stress and inflammation, and hepatic inflammation in C57BL/6 mice [9].

**PS exposure induces systematic metabolic dysfunctions involved in gut microbial enrichment**

Metagenomics indicated that 31 KEGG (Kyoto Encyclopedia of Genes and Genomes) pathways at level 3 were significantly altered upon PS exposure, and that 968 KEGG genes, involved in 333 pathways, were significantly altered, among which 269 genes were linked to metabolic pathways (Figures S7A and S7B). The overview of metabolic pathways showed significant changes in microbial metabolism, such as those in oxidative phosphorylation, fatty acid metabolism, and central carbon metabolism (Figure S7C). Moreover, 38 pathways were significantly enriched upon PS exposure, among which 35 pathways were involved in metabolism (Figure S7D). These data indicated systematic metabolic dysfunctions during PS‐induced gut microbial enrichment at the gene level.

Metabolomics revealed that the metabolic profile of gut microbiota was significantly altered upon PS exposure, and that 88.39% of 155 differential metabolites were significantly decreased (Figure S8). Systematic decreases in differential metabolites suggested potential decreases in the metabolic activity of gut microbiota upon PS exposure, which is consistent with PS‐induced gut microbial enrichment. Notably, some metabolites involved in nervous and/or hormonal functions were increased in gut microbiota under PS exposure, such as phenylethylamine, serotonin, deoxycorticosterone, thyroxine, 17‐hydroxypregnenolone sulfate, and enterolactone (Figure S9A). Totally, 27 metabolic pathways (mainly involved in redox homeostasis, nervous and hormonal functions) were significantly altered after PS exposure (Figure S9B). Furthermore, many changes in the above pathways could be found in metagenomic data. Accordingly, PS‐induced changes in pathways linked to redox homeostasis, nervous, and hormonal dysfunctions were further explored.

**PS exposure induces metabolic dysfunctions involved in redox homeostasis in gut microbiota**

**Figure 2:**
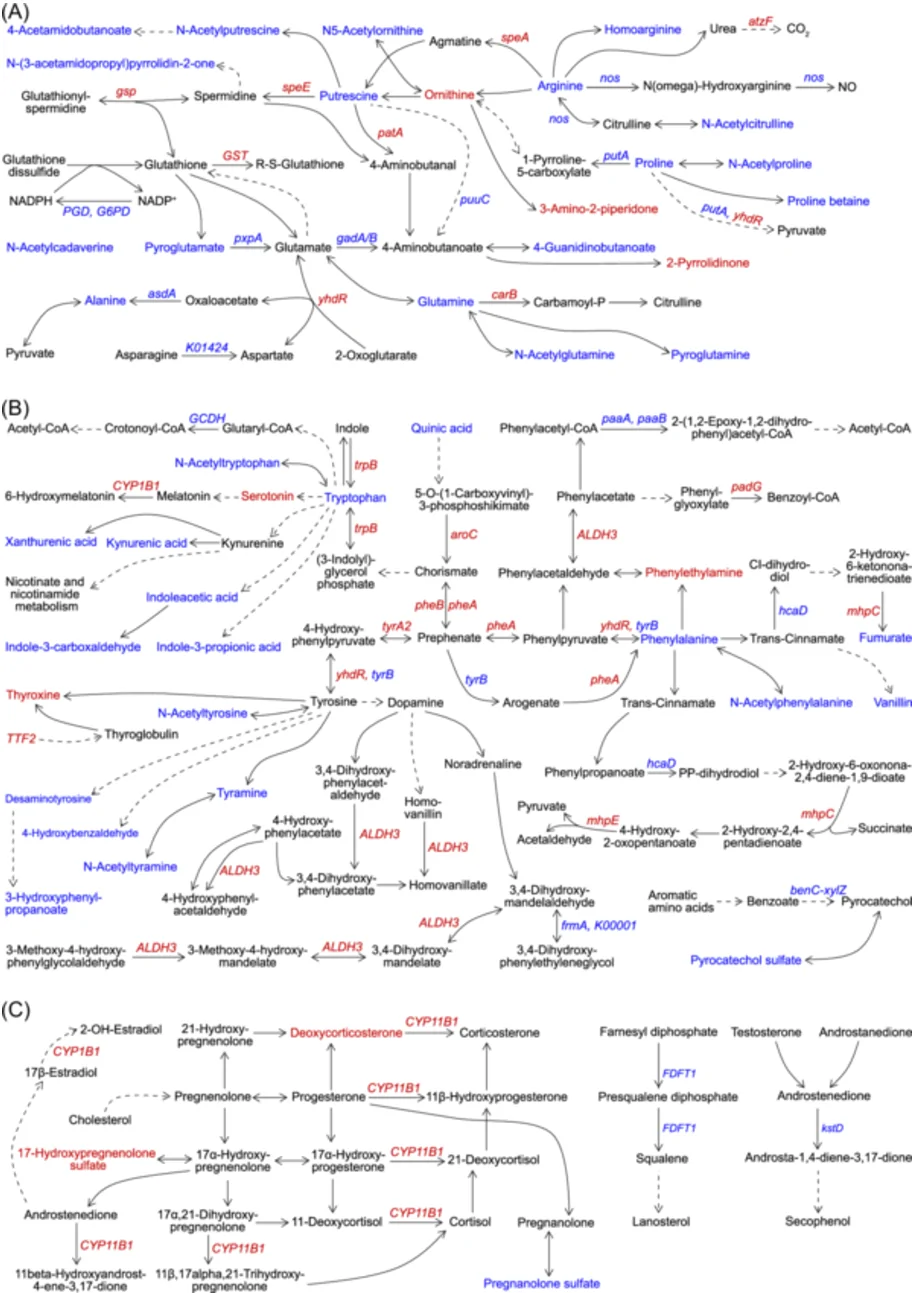
Polystyrene microplastic (PS) exposure induces metabolic dysfunctions involved in redox homeostasis, nervous and hormonal functions in gut microbiota. (A) Changes in glutathione metabolism, arginine and proline metabolism, arginine biosynthesis, and alanine, aspartate, and glutamate metabolism. (B) Changes in aromatic amino acid metabolism. (C) Changes in steroid metabolism. Red/blue fonts: significantly increased/decreased upon PS exposure (*p* < 0.05, two‐sided Mann–Whitney *U* test). Dashed arrows indicate that conversions between metabolites require at least two steps of biochemical reactions. Solid arrows indicate that biochemical conversions can occur directly between metabolites.

Glutathione and related amino acid metabolism were altered in gut microbiota exposed to PS (Figure 2A and Figure S10A). *PGD* and *G6PD* were decreased in gut microbiota exposed to PS, which suggested less NADPH generation for restoring oxidative glutathione to reductive glutathione. Interestingly, *speA*, *speE*, *gsp*, and *GST* were increased, which indicated increases in spermidine biosynthesis from putrescine, glutathionylspermidine synthesis from glutathione and spermidine, glutathionylspermidine hydrolysis into glutathione and spermidine, and glutathionylation of proteins, xenobiotics, and/or other substances under PS exposure. Moreover, pyroglutamate, glutamine, alanine, *pxpA*, *gad*, and *asdA* decreased, indicating metabolic disturbances in degradation products and precursors of glutathione under PS exposure. These data demonstrated the involvement of glutathione and related amino acid metabolism in maintaining redox homeostasis in gut microbiota under PS exposure, especially the biosynthesis and hydrolysis of glutathionylspermidine, which is more effective than glutathione in preventing reactive oxygen species (ROS)‐induced damages in DNAs and proteins [10, 11]. Gut microbiota might use the spermidine synthase to generate excess spermidine for host invasion, concurrently producing glutathionylspermidine and 5'‐methylthioadenosine as defense against external pressure, such as oxidative stress and inflammation derived from pollutant exposure and/or host defense system [11, 12].

Systematic decreases in pathways involved in energy production also occurred in gut microbiota under PS exposure (Figure S10B). Citrate, cis‐aconitate, fumarate, *OGDH*, and *frdC* were decreased, while *K01677*, *K01678*, *K05942*, *porA/C/D*, *K01596*, *oadA*, and *aceB* were increased under PS exposure, suggesting a decrease in the oxidative tricarboxylic acid cycle to provide biological energies, and increases in the reductive tricarboxylic acid cycle and gluconeogenesis. Consistently, most genes involved in oxidative phosphorylation were decreased upon PS exposure, demonstrating decreases in oxidative phosphorylation and resultant generation of ROS and energy. Besides, most genes involved in the degradation of fatty acids, aromatic compounds, and/or benzoate were decreased under PS exposure, suggesting decreases in the resultant generation of ROS and energy from the above pathways. Notably, metabolic functions of plastisphere microbiota are typically decreased, while the health risks (such as selective enrichment of pathogenic and/or drug‐resistant microbes) are increased [13]. This phenomenon might be linked to fitness costs of plastisphere microbiota to cope with adverse environments.

**PS exposure induces metabolic disturbances involved in nervous and hormonal dysfunctions in gut microbiota**

Metabolic disturbances related to PS‐induced nervous and hormonal dysfunctions in gut microbiota were discovered (Figure 2B,C, and Figure S11). Tryptophan, N‐acetyltryptophan, kynurenate, xanthurenate, indoleacetate, indole‐3‐carboxaldehyde, and indole‐3‐propionate were decreased, while serotonin and *CYP1B1* were increased in gut microbiota under PS exposure, which indicated decreases in the tryptophan‐kynurenine pathway and tryptophan‐indole pathway, and an increase in the tryptophan‐serotonin pathway. Regarding tyrosine metabolism, *aroC*, *pheA*, *pheB*, *tyrA2*, *yhdR*, *TTF2*, *ALDH3*, and thyroxine were increased, whereas *tyrB*, N‐acetyltyrosine, tyramine, N‐acetyltyramine, desaminotyrosine, 3‐hydroxyphenylpropanoate, and 4‐hydroxybenzaldehyde were decreased in gut microbiota under PS exposure, suggesting the activation of tyrosine‐thyroxine pathway and dopamine degradation. Besides, *hacD*, phenylalanine, N‐acetylphenylalanine, and vanillin were decreased, whereas *ALDH3*, *padG*, *mhpC*, *mhpE*, and phenylethylamine were increased in gut microbiota under PS exposure, which suggested an increase in the phenylalanine‐phenylacetate catabolic pathway. Moreover, pathways related to neurological functions were also altered under PS exposure, demonstrating the involvement of gut microbiota in PS‐induced neurological dysfunctions (Figure S11). Studies showed that microplastic exposure disrupted the blood‐brain barrier, lowered the dendritic spine density, and induced inflammation in the hippocampus and cognitive and memory deficits in mice [2, 14].

Disorders of steroid metabolism in gut microbiota under PS exposure were found as well (Figure 2C). Concerning the steroid hormone biosynthesis pathway, deoxycorticosterone, 17‐hydroxypregnenolone sulfate, *CYP11B1*, and *CYP1B1* were increased, whereas pregnanolone sulfate was decreased in gut microbiota under PS exposure. Meanwhile, *FDFT1* and *kstD*, separately involved in de novo steroid synthesis and steroid degradation, were decreased in gut microbiota exposed to PS. These data suggested that the accumulation of deoxycorticosterone and 17‐hydroxypregnenolone sulfate was probably due to cholesterol‐derived steroid synthesis. Pregnanolone sulfate, deoxycorticosterone, and 17‐hydroxypregnenolone sulfate mediate the activity of ligand‐gated ion channel‐associated receptors (e.g., γ‐aminobutyric acid receptor, N‐methyl‐d‐aspartate receptor) by themselves or their metabolites, thus affecting the pathophysiology of psychiatric disorders, such as schizophrenia, anxiety, and depression [15, 16]. Moreover, enterolactone and enterodiol, with antioxidant, anti‐inflammatory, estrogenic, and anti‐estrogenic activities, were significantly altered in gut microbiota under PS exposure in this study [17, 18].

**Species contributions to metabolic dysfunctions during gut microbial enrichment under PS exposure**

We found that KEGG pathways were significantly associated with gut microbiota from the class to species level, and that Firmicutes and Bacteroidota were the top two phyla that contributed most to KEGG pathways (Figures S12 and S13). Among dominant contributing species to KEGG pathways, contributions of Oscillospiraceae bacterium and Clostridiales bacterium were significantly increased under PS exposure (Figure S14). Notably, 80.29% of species contributions from bacteria within Firmicutes were significantly increased in gut microbiota under PS exposure, whereas 97.25% of the above species contributions from bacteria within Bacteroidota were decreased (Figures S15 and S16). Increases in species contributions to KEGG pathways under PS exposure mainly included those of Oscillospiraceae bacterium, Clostridiales bacterium, *Clostridia bacterium*, Lachnospiraceae bacterium, and *Clostridium* sp., whereas decreases in species contributions to KEGG pathways under PS exposure mainly included those of *Bacteroides acidifaciens* and *Aerococcus urinaeequi* (Figures S15−S17). These data highlighted species contributions of Firmicutes to metabolic functions under PS exposure, especially Eubacteriales, such as Oscillospiraceae bacterium and Clostridiales bacterium.

## 2 CONCLUSION

In this study, we deciphered microplastic‐induced distinctive gut microbial enrichment, which was dominated by Eubacteriales. At this point, the gut microbial polyamine synthetic pathway was activated to maintain glutathionylspermidine homeostasis. Besides, pathways related to energy and ROS production were decreased. Moreover, disturbances in aromatic amino acid metabolism were observed in gut microbiota. This study provides novel perspectives and potential intervention targets for investigations on the health effects of microplastics on gut microbiota and functions, as well as the host. Novel discoveries and potential intervention targets (e.g., Eubacteriales enrichment, increased polyamine‐driven glutathionylspermidine homeostasis, cholesterol‐derived hormone synthesis, and the tryptophan‐serotonin pathway under PS exposure) were summarized in the graphic abstract.

## 3 METHODS

Detailed procedures for animal experiments, hematoxylin and eosin staining, metagenomic analysis, metabolomic analysis, and statistical analysis are available in the Supplementary Information.

## Supporting information

Supplementary Material.

## Data Availability

Raw sequencing data have been uploaded to the Science Data Bank (https://www.scidb.cn/en/s/YrYnI3). The data used are saved in GitHub (https://github.com/yeguozhu1983/Microplastic-induced-gut-microbial-enrichment-and-dysfunctions). Supplementary materials (methods, figures, graphical abstract, data sources, slides, videos, Chinese translated version, and update materials) may be found in the online DOI or iMetaOmics http://www.imeta.science/imetaomics/. The data that support the findings of this study are openly available in the Science Data Bank at https://www.scidb.cn/en/s/YrYnI3.
