# Supplementary material for: Distinctive metabolic disturbances associated with redox homeostasis, nervous and hormonal functions during gut microbial enrichment upon polystyrene microplastic exposure
Source: IMetaOmics. 2025 Jul 23;2(4):e70043. doi: 10.1002/imo2.70043 (PMC12806064; doi:10.1002/imo2.70043)
Supplement: Supplementary file 1 — Supplementary Material. [file IMO2-2-e70043-s001.doc]

Supporting information to **Distinctive metabolic disturbances associated with redox homeostasis, nervous and hormonal functions during gut microbial enrichment upon polystyrene microplastic exposure**

**Running title**: Gut microbial enrichment and dysfunctions upon microplastic exposure

Guozhu Ye^1,#,*^, Zeming Wu^2,#^, Guoyou Chen^3^, Xuyi Liu^1^, Yifang Duan^1^, Minghui Li^3^, Qiansheng Huang^1^

^1^Xiamen Key Laboratory of Indoor Air and Health, State Key Laboratory for Ecological Security of Regions and Cities, State Key Laboratory of Advanced Environmental Technology, Institute of Urban Environment, Chinese Academy of Sciences, Xiamen 361021, China.

^2^iPhenome Biotechnology (Dalian) Inc., 36 Xingxian Road, High-tech zone district, Dalian 116085, China.

^3^College of pharmacy, Daqing Campus, Harbin Medical University, Daqing, 163319, China.

^#^These authors contributed equally: Guozhu Ye, Zeming Wu.

^*^Correspondence: [gzye@iue.ac.cn](mailto:gzye@iue.ac.cn) (Guozhu Ye).

**MATERIALS AND METHODS**

**Animal experiments and sample collection**

All animal experiments in this study were conducted in line with the Guidelines for the Care and Use of Laboratory Animals and the ARRIVE (Animal Research: Reporting of In Vivo Experiments) guidelines, and approved by the Animal Ethics Committee of Harbin Medical University-Daqing (approval number, HMUDQ20240110011) [1]. Sprague Dawley rats (male, 6 weeks old) were obtained from Changchun Yisi Laboratory Animal Technology Co., Ltd. (Changchun, China). Rats were housed in a specific pathogen-free facility with a temperature of 20 ± 2 °C, a 12-h light-dark cycle, and a relative humidity of 60 ± 10%. After one week of acclimatization, rats are randomly assigned to the control and PS exposure group (n = 8 each group).

It was revealed by pyrolysis–gas chromatography–mass spectrometry analysis that the average content of polyethylene and polyvinyl chloride microplastics in patients with carotid artery plaques could separately reach 21.7 ± 24.5 and 5.2 ± 2.4 μg/mg plaques [2]. Meanwhile, the maximum level of polyethylene and polyvinyl chloride microplastics were separately about 150 and 11 μg/mg plaques in patients with carotid artery plaques [2]. Besides, the content of microplastics ingested by each person per day can be 0.01–66.81 g [3,4]. Assuming that a person weighs 60 kg, the daily amount of microplastics ingested by a person is 0.17-1113.5 mg/kg. Microplastics with a particle size of less than 10 µm (especially below 5 µm) are an important component of microplastics in the environment and the human body [2,5−14]. Our previous works showed that 2-, 10-, and 200-μm PS exposure separately triggered lipid accumulation, damages, and/or inflammation in the liver of marine medaka (*Oryzias melastigmas*), and caused dysbiosis and dysfunctions in the gut and water microbiota [15−17]. It is well known that small microplastics are more likely to pass through the gut barrier than larger microplastics, entering the circulatory system and accumulating in organs, which can lead to inflammation, oxidative stress, and resultant more serious toxic effects [2,8,16−18]. Moreover, exposure to microplastics for 28 or 30 days or even less can induce toxic effects, such as microplastic accumulation in organs, inflammation, oxidative stress, metabolic disorders, and gut microbial dysbiosis [19−23].

Accordingly, rats in the exposure group were treated with 25 mg/kg of 2-µm PS (Figure S1) daily by gavage for 28 days, while rats in the control group were daily given an equal volume of deionized water by gavage for 28 days. After the treatment, 4 rats were randomly selected from the control and exposure group respectively. After the microplastic exposure, 4 rats were randomly selected from the control and exposure group respectively. Rats were placed alone in the metabolic cage, and allowed to defecate freely. The stool sample of each rat was collected individually and immediately by using sterile forceps, and stored in a sterile tube at − 80 °C for following metabolomic and metagenomic analysis.

**Hematoxylin and eosin staining of colonic tissues**

Colonic tissues of rats were collected, and fixed with 4% paraformaldehyde solution. Following cutting into 4-μm sections, the tissue was embedded with paraffin. After that, the tissue was cut into multiple sections, and stained with hematoxylin and eosin for morphological observations.

**Metagenomic analysis**

Microbial DNA from the stool sample (about 0.3 g) was extracted by the Fast DNA Stool Mini Kit (Qiagen, Germany). The Illumina platform was used for DNA library construction and sequencing at Majorbio Co., Ltd. (Shanghai, China). Raw sequencing reads were processed by the fastp tool (version 0.20.0) [24]. After the quality control and elimination of the host DNA sequence, 93.64 Gb of data from all samples was obtained, with an average of 11.70 Gb per sample. To eliminate the host DNA contamination, reads were mapped to the rat genome (rat_ncbi_Rattus_norvegicus.Rnor_6.0.dna.chromosome) using BWA (version 0.7.17, http://bio-bwa.sourceforge.net). Clean reads were assembled by the assembler MEGAHIT (version 1.1.2) [25]. Contigs with the minimum length of 300 bp were selected and used for the prediction of open reading frames by the program Prodigal (version 2.6.3) [26]. The open reading frame with a minimum length of 100 bp was selected and translated into amino acid sequences. The gene sequences were clustered by the program CD-HIT to obtain non-redundant gene sets and their base sequences [27]. High-quality reads of each sample were compared with the non-redundant gene set by the software SOAPaligner (version soap2.21 release, https://github.com/ShujiaHuang/SOAPaligner). Gene abundances were calculated as (Reads Number/Gene Length)_Relative [28]. The software DIAMOND (version 2.0.13, key parameters: BLASTP, e-value ≤ 1E-5) was employed for gene-based taxonomic annotations of species (from domain to species) according to the NCBI-NR database (version NR 202209) [29]. Besides, the software DIAMOND was used for the gene alignment to KEGG databases (version 202209). After that, the gene functional annotation was conducted via KEGG Orthology Based Annotation System 2.0 [30].

**Metabolomic analysis**

After the stool sample was lyophilized, weighed (about 20 mg), and put in a centrifuge tube, 1000 µL of 80% methanol was added. The sample was homogenized by the high-throughput tissue grinder SCIENTZ-12 (Ningbo Xinzhi Biotechnology Co., Ltd, China) at 1,500 rpm for 3 min. After vortex mixing, 600 µL of the homogenate was absorbed into a new centrifuge tube and centrifuged at 13,000 g for 15 min. Subsequently, 200 µL of the supernatant was lyophilized and stored at -80 °C for subsequent metabolomic analysis. Quality control samples were prepared by mixing an equal volume of the homogenate from each sample and processed in the same way as other analytical samples.

Following dissolution, the sample was submitted for metabolomics analysis by an Ultimate^TM^ 3000 UPLC coupled with Q Exactive^TM^ quadrupole-Orbitrap mass spectrometer system (Thermo Scientific, San Jose, USA) according to our previous methods with minor modifications [31]. The metabolite profiling was acquired by the reverse phase chromatographic separation mode coupled with positive and negative ionization detection, respectively. The injection volume, flow rate of the mobile phase, and column temperature were set to the same parameters for the positive and negative ionization detection: 5 μL, 0.4 mL/min, and 50 °C, respectively. During the positive detection, an ACE C_18_-PFP column (Advanced Chromatography Technologies Ltd, Aberdeen, Scotland) was employed to separate metabolites. Acetonitrile and water with 0.1% formic acid were separately employed as the mobile phase. The elution gradient started from 2 to 98% of the organic mobile phase in 10 min. For the negative detection, an Acquity^TM^ HSS C_18_ column for (Waters Corporation, Milford, USA, 1.8 μm, 2.1 × 100 mm) was used to separate metabolites. Water and acetonitrile/methanol (4/1, v/v), both containing 6 mM ammonium bicarbonate buffer salt, were applied as the mobile phase. The elution gradient started from 2 to 100% of the organic phase in 10 min, and was maintained for 5 min to wash the column and equilibrate the system.

Identical ionization parameters, except the ionization voltage on the quadrupole-Orbitrap mass spectrometer were operated: heater temperature, 355 °C; capillary temperature, 320 °C; aux gas, 10 arb; sheath gas, 45 arb; and S-Lens RF, 55%. Mass spectrometry signals of metabolites were acquired in the full scan mode using the parameters: maximum injection time, 200 ms; automatic gain control target, 1E6; and mass resolution, 70,000 (full width at half maximum). The mass scanning range of 70-1000 (m/z) was employed. Repeated injection of quality control samples were conducted to obtain the top 10 data-dependent MS^2^ spectra for further structural annotation of metabolites. The mass resolution of 17,500 (full width at half maximum) was employed for full acquisition of MS/MS data. Isotope exclusion, dynamic exclusion and apex trigger were performed. The precursor isolation window was 1.0 Da. Stepped normalized collision energy was applied for the collision-induced dissociation of metabolites, where the ultra-pure nitrogen was the fragmentation gas. The data were acquired in the profile format.

Compound Discoverer software (Thermo Scientific, San Jose, USA) was employed to resolve the full scan data and data-dependent MS^2^ data to obtain metabolite information as much as possible. Metabolites were preliminarily annotated by comparing the acquired structural information of MS^2^ against that in mzCloud library (Thermo Scientific, San Jose, USA) and a proprietary iPhenome^TM^ SMOL high resolution MS/MS spectrum local library created using authentic standards. Meanwhile, the MS^1^ spectra were compared against those in a local HMDB database, and the mass tolerance was 5 ppm [32]. Moreover, metabolite annotation was further confirmed based on the retention time and elution order of metabolites on the column and the similarity of the high-resolution MS/MS spectra. Finally, the annotation results and identification levels were determined according to the metabolomics standards initiative [33].

**Statistical analysis**

Principal component analysis, partial least squares-discriminant analysis, and pathway analysis were performed via MetaboAnalyst 6.0 [34]. The two-sided Mann-Whitney *U* test and heat map plot were conducted by MeV 4.9.0 [35]. After the abundances of each microbe was mean-centered and then divided by its standard deviation, the data were employed for the heat map plot. Analysis of the Chao1 index, principal coordinate analysis, regression analysis, and species contributions to KEGG functions were executed by the Majorbio cloud platform. The Chao1 estimator (https://mothur.org/wiki/calculators/) was used to calculate the richness of gut microbiota. The first principal component from principal component analysis was used for the regression analysis to evaluate associations of changes in viruses with those in other microbes, where data on viruses and other microbes were separately employed as the independent and dependent variables. Besides, the first principal component from principal coordinate analysis was employed for the regression analysis to determine associations of changes in KEGG pathways at level 3 with those in gut microbiota from the phylum to species level, where data on KEGG pathways at level 3 and gut microbiota were separately employed as the independent and dependent variable. The KEGG mapper was used for the pathway mapping of differential genes (https://www.kegg.jp/kegg/mapper/color.html). The statistically significant level was lower than 0.05.


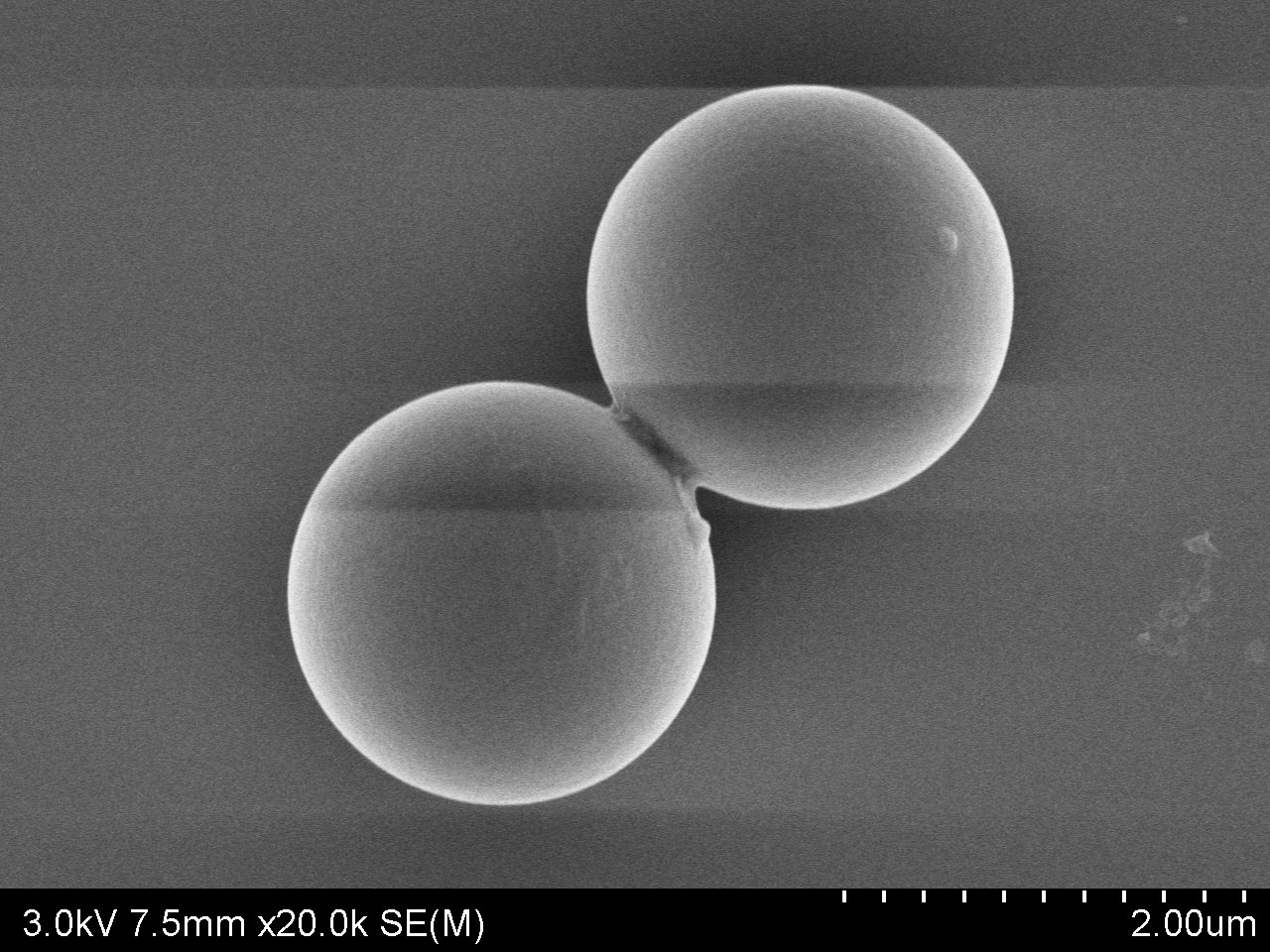


Figure S1 The morphology and size of polystyrene microplastics.


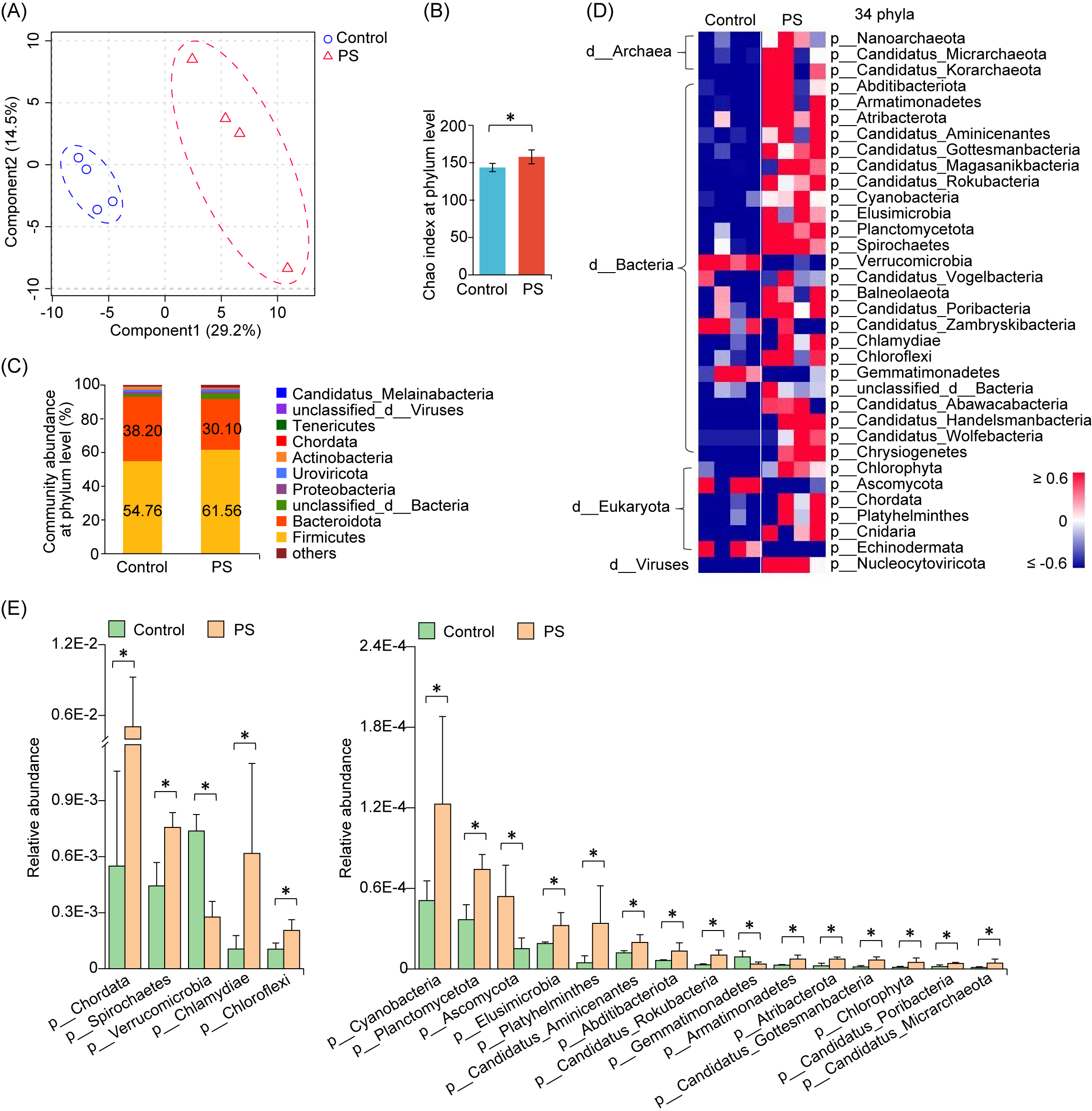


Figure S2 Polystyrene microplastic (PS) exposure induces gut microbial enrichment at the phylum level. (A) Partial least squares-discriminant analysis of PS-induced changes in gut microbiota. (B) PS-induced changes in the chao index. (C) PS-induced changes in the microbial composition. (D) Heat map plot of PS-induced changes in gut microbiota. Microbes significantly altered (*p* < 0.05, two-sided Mann-Whitney *U* test) upon PS exposure were all listed. (E) Column plot of PS-induced changes in gut microbiota. The top 20 most abundant microbes with significant alterations upon PS exposure were listed. *, *p* < 0.05, two-sided Mann-Whitney *U* test. The average plus standard deviation was employed for the column plot.


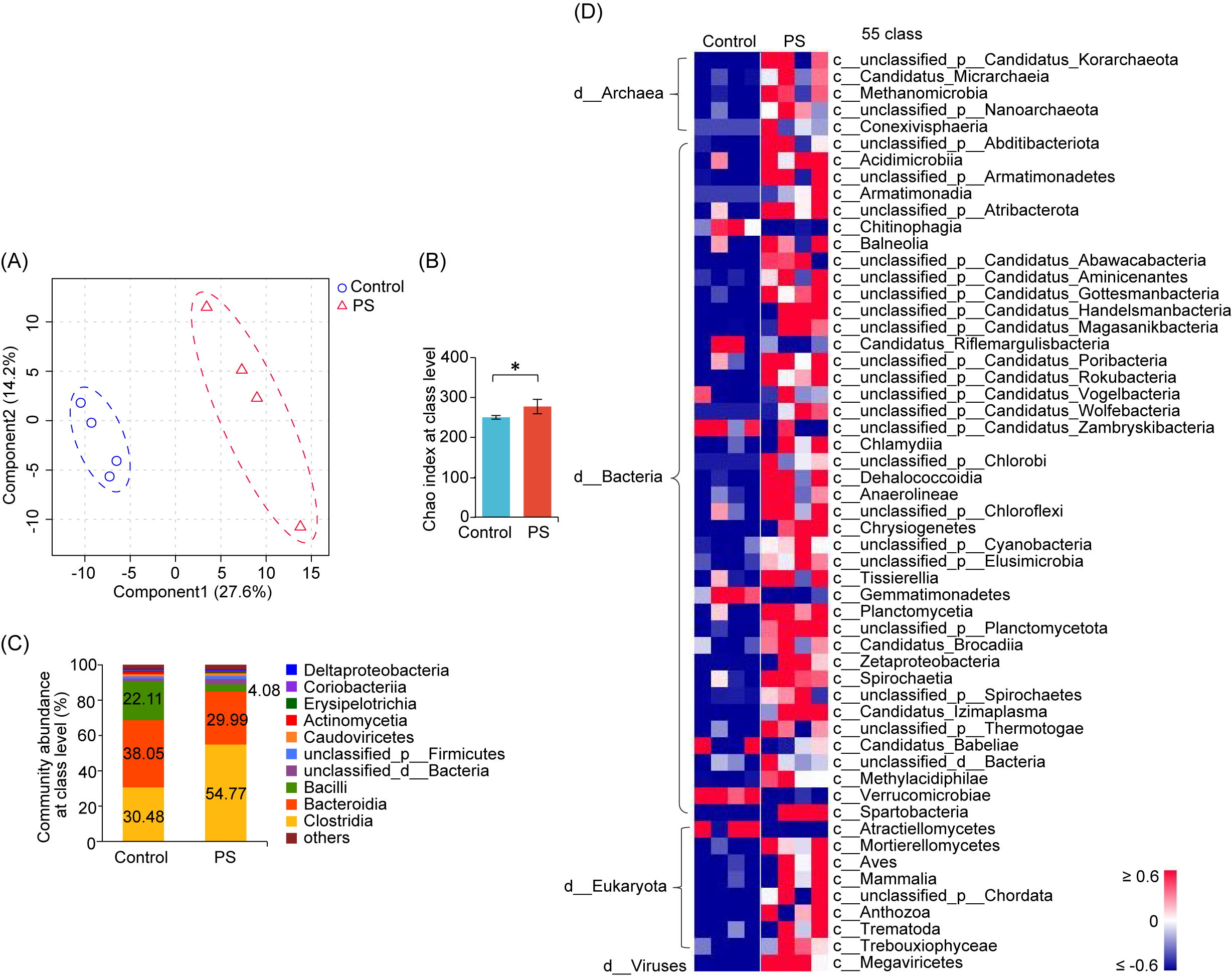


Figure S3 Polystyrene microplastic (PS) exposure induces gut microbial enrichment at the class level. (A) Partial least squares-discriminant analysis of PS-induced changes in gut microbiota. (B) PS-induced changes in the chao index. (C) PS-induced changes in gut microbial composition. (D) Heat map plot of PS-induced changes in gut microbiota. Microbes significantly altered (*p* < 0.05, two-sided Mann-Whitney *U* test) upon PS exposure were all listed.


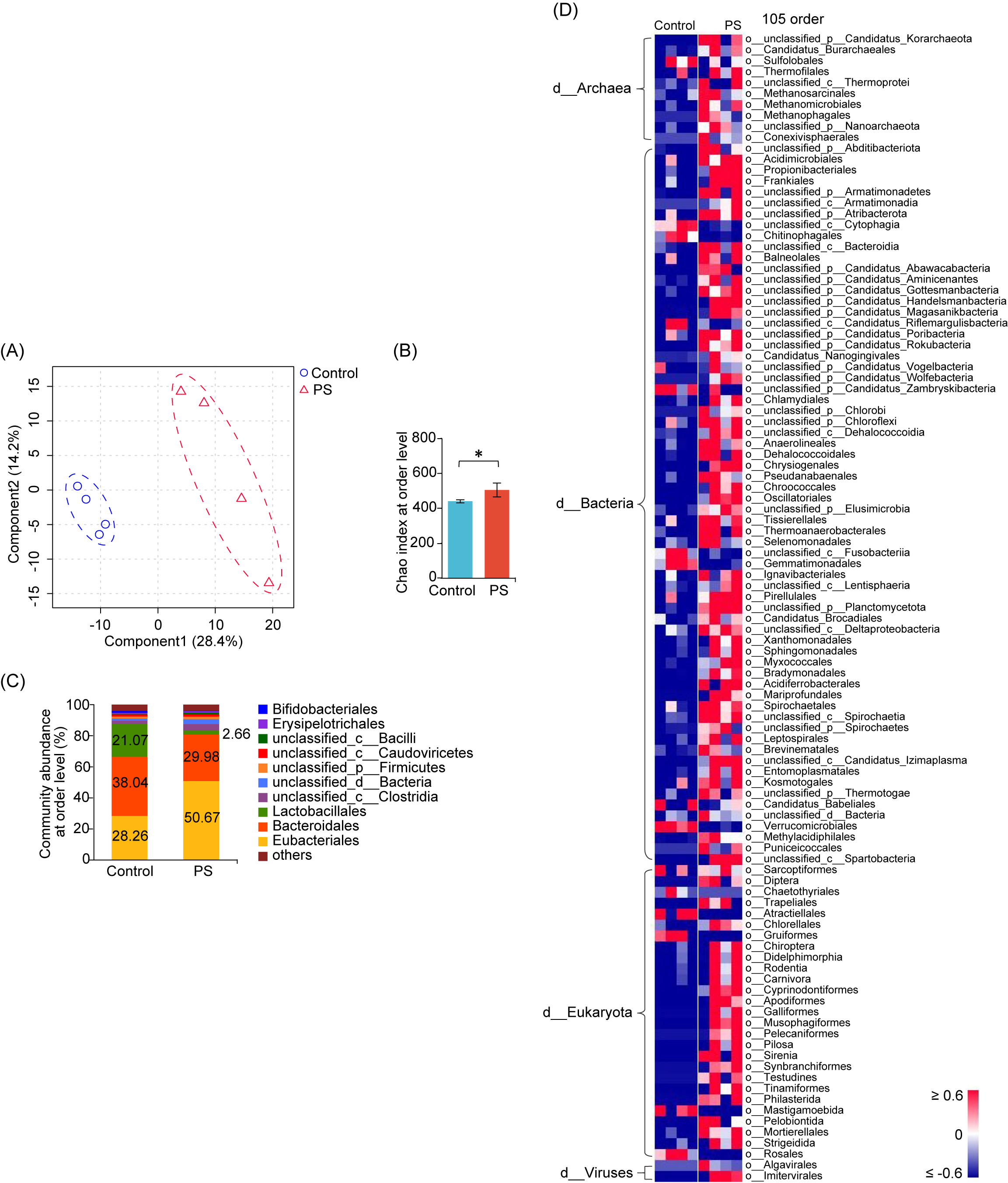


Figure S4 Polystyrene microplastic (PS) exposure induces gut microbial enrichment at the order level. (A) Partial least squares-discriminant analysis of PS-induced changes in gut microbiota. (B) PS-induced changes in the chao index. (C) PS-induced changes in gut microbial composition. (D) Heat map plot of PS-induced changes in gut microbiota. Microbes significantly altered (*p* < 0.05, two-sided Mann-Whitney *U* test) upon PS exposure were all listed.


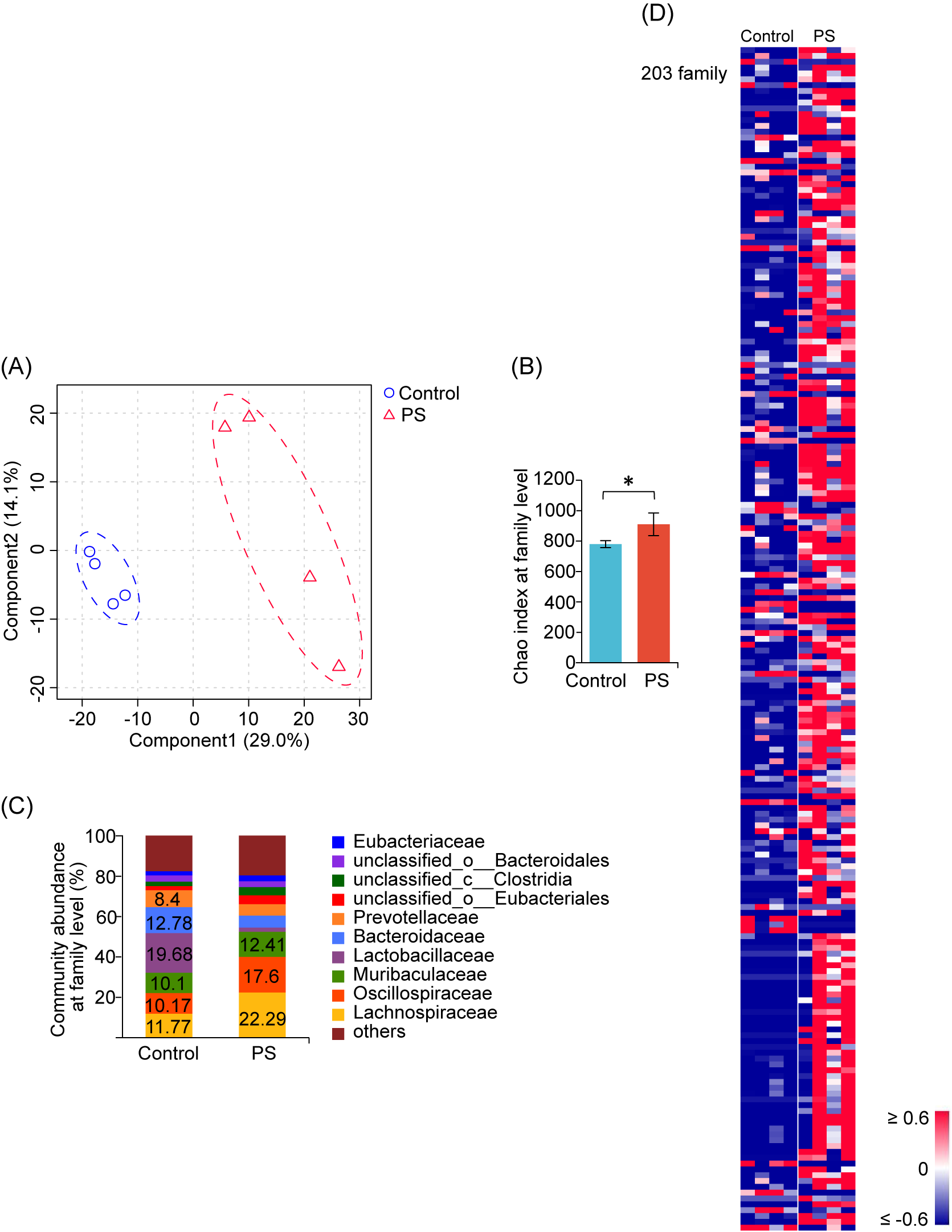


Figure S5 Polystyrene microplastic (PS) exposure induces gut microbial enrichment at the family level. (A) Partial least squares-discriminant analysis of PS-induced changes in gut microbiota. (B) PS-induced changes in the chao index. (C) PS-induced changes in gut microbial composition. (D) Heat map plot of PS-induced changes in gut microbiota. Microbes significantly altered (*p* < 0.05, two-sided Mann-Whitney *U* test) upon PS exposure were all listed.


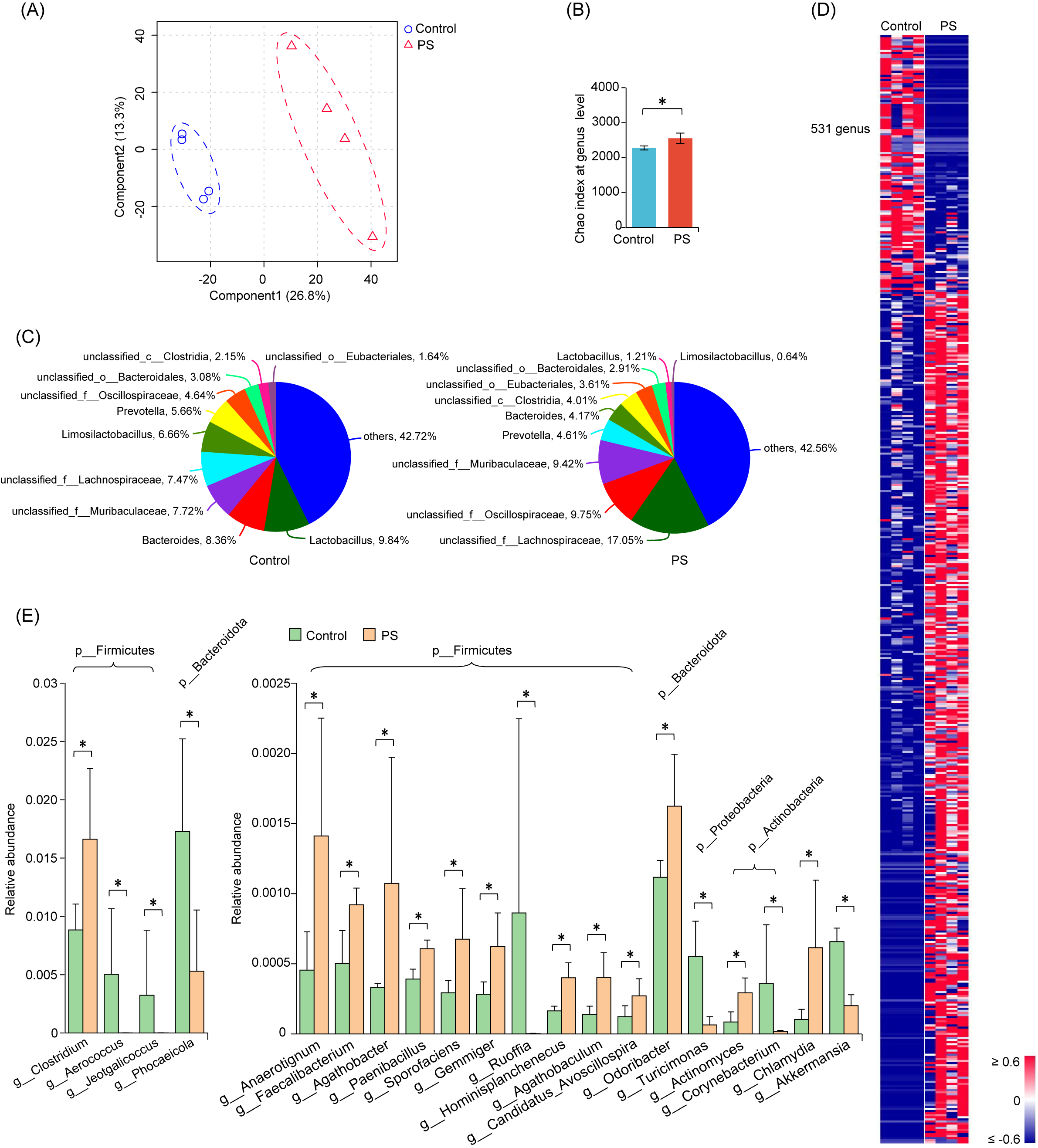


Figure S6 Polystyrene microplastic (PS) exposure induces gut microbial enrichment at the genus level. (A) Partial least squares-discriminant analysis of PS-induced changes in gut microbiota. (B) PS-induced changes in the chao index. (C) PS-induced changes in the microbial composition. (D) Heat map plot of PS-induced changes in gut microbiota. Microbes significantly altered (*p* < 0.05, two-sided Mann-Whitney *U* test) upon PS exposure were all listed. (E) Column plot of PS-induced changes in gut microbiota. The top 20 most abundant microbes with significant alterations upon PS exposure were listed. *, *p* < 0.05, two-sided Mann-Whitney *U* test. The average plus standard deviation was employed for the column plot.


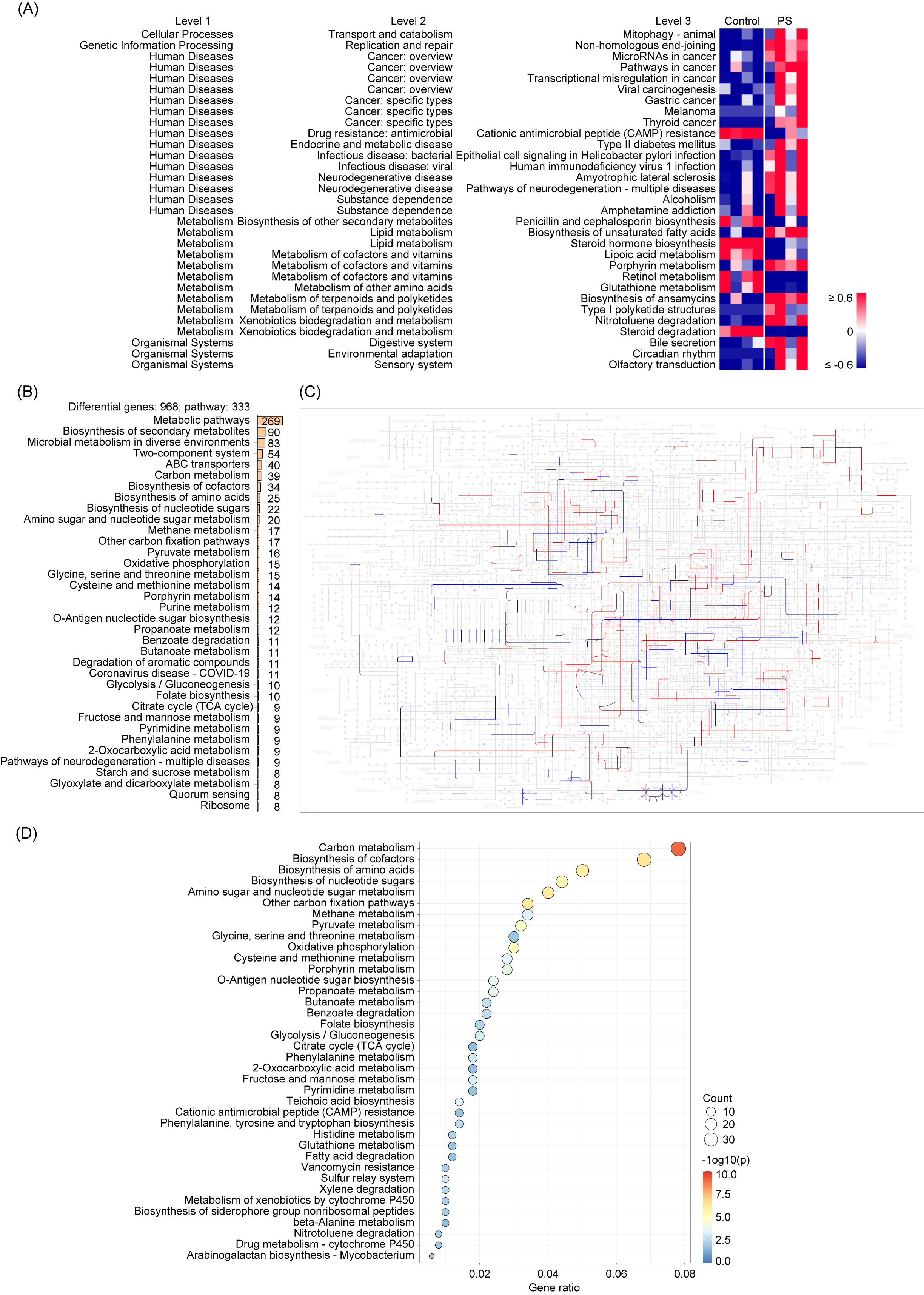


Figure S7 Polystyrene microplastic (PS) exposure induces metabolic dysfunctions in gut microbiota at the gene level. (A) Pathways significantly altered (*p* < 0.05, two-sided Mann-Whitney *U* test) at the pathway level. (B) The top 36 pathways enriched with the highest number of differential genes (*p* < 0.05, two-sided Mann-Whitney *U* test). (C) Pathway mapping of differential genes to metabolic pathways. Red/blue lines, genes significantly increased/decreased upon PS exposure. (D) Pathway enrichment analysis of differential genes.


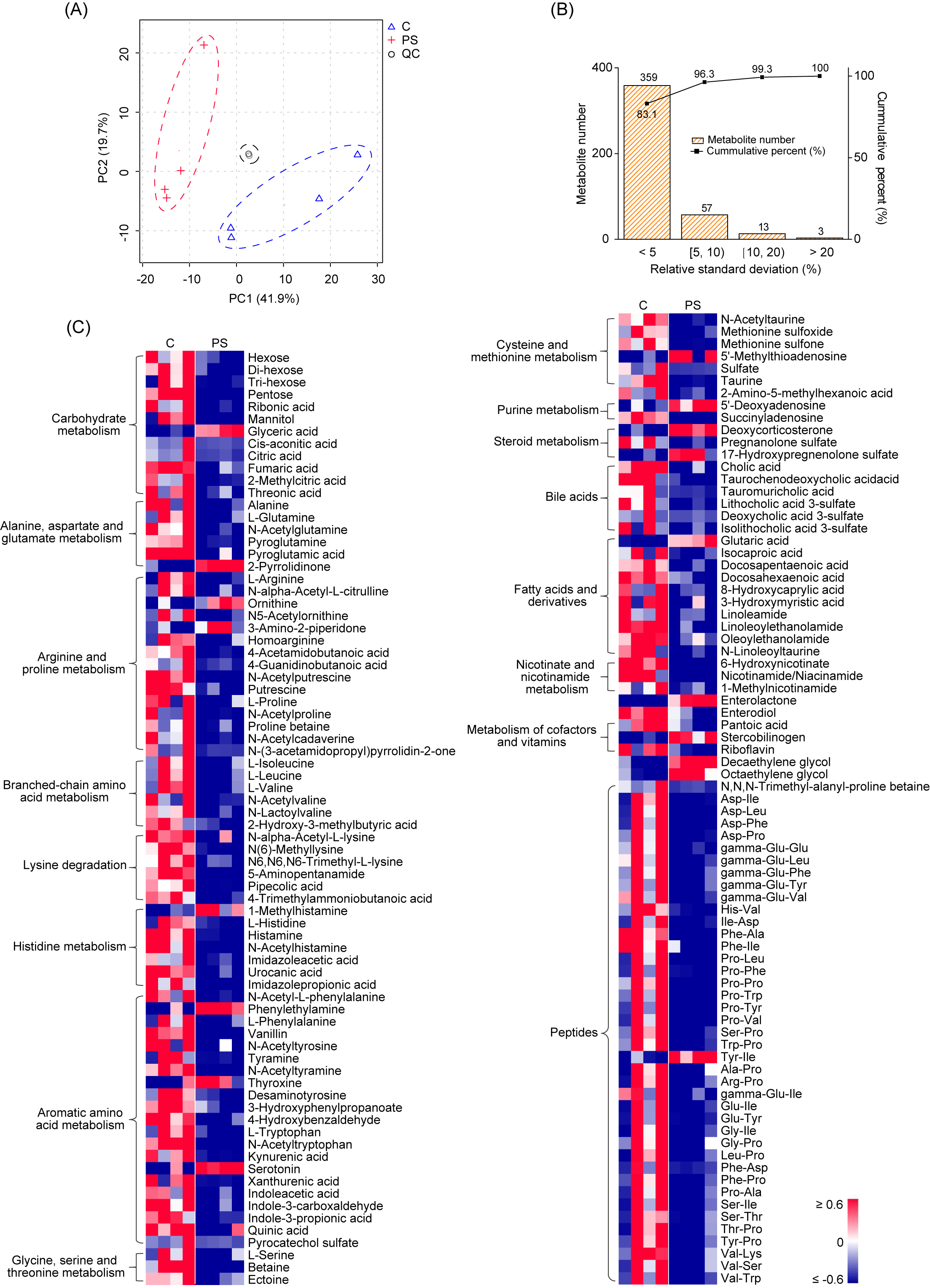


Figure S8 Polystyrene microplastic (PS) exposure induces metabolic dysfunctions in gut microbiota at the metabolite level. (A) Principal component analysis of changes in the metabolic profiling of gut microbiota upon PS exposure. QC, quality control samples. (B) Relative standard deviation distribution of metabolites in quality control samples. (C) The heat map plot of PS-induced metabolic dysfunctions. All differential metabolites (*p* < 0.05, two-sided Mann-Whitney *U* test) were listed. C, the control group.


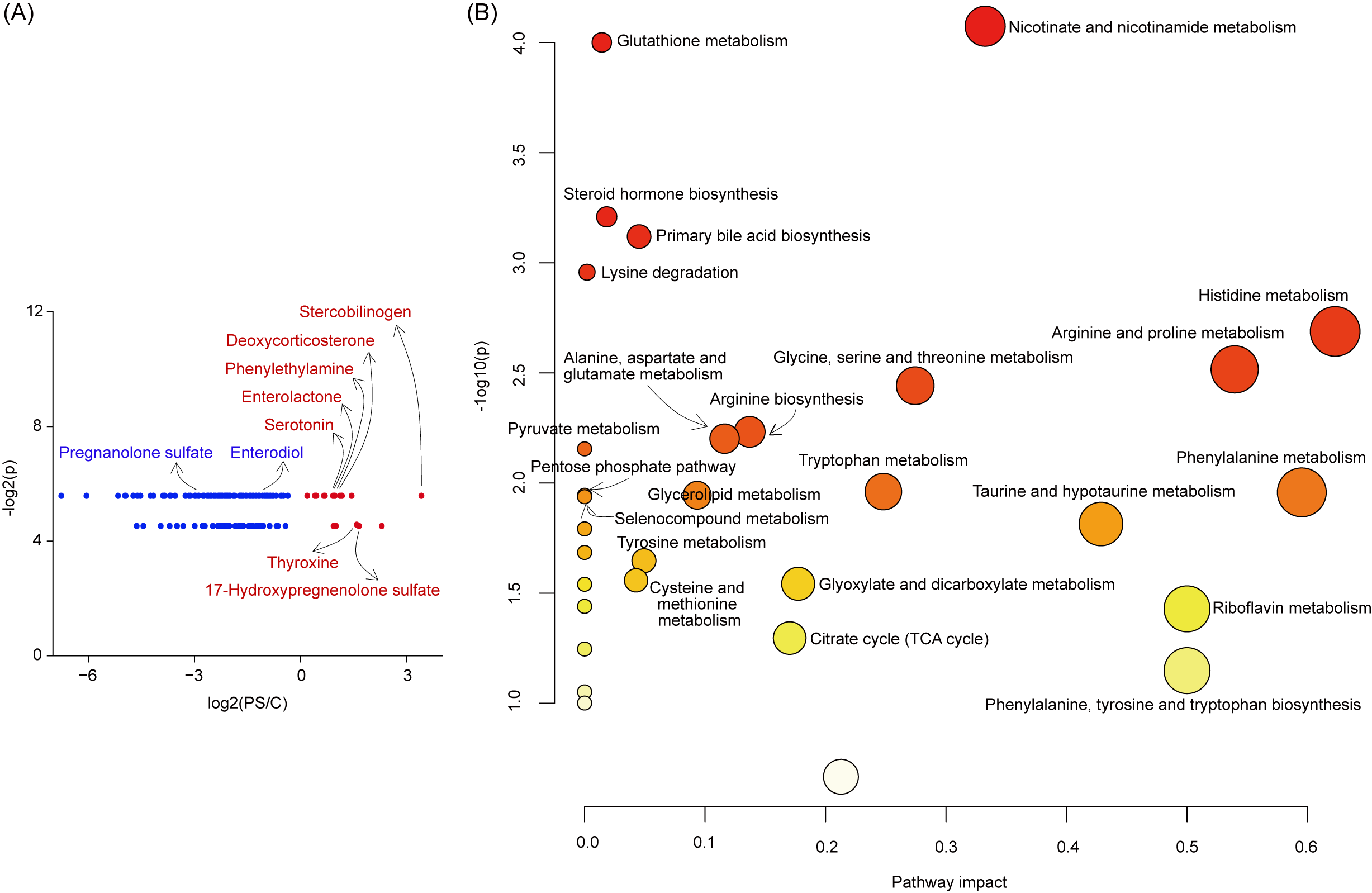


Figure S9 Changes in major metabolic pathways induced by polystyrene microplastic (PS) exposure in gut microbiota at the metabolite level. (A) The volcano plot of PS-induced changes in metabolites. Only differential metabolites (*p* < 0.05, two-sided Mann-Whitney *U* test) were listed. (B) Pathway analysis of PS-induced changes in metabolic pathways.


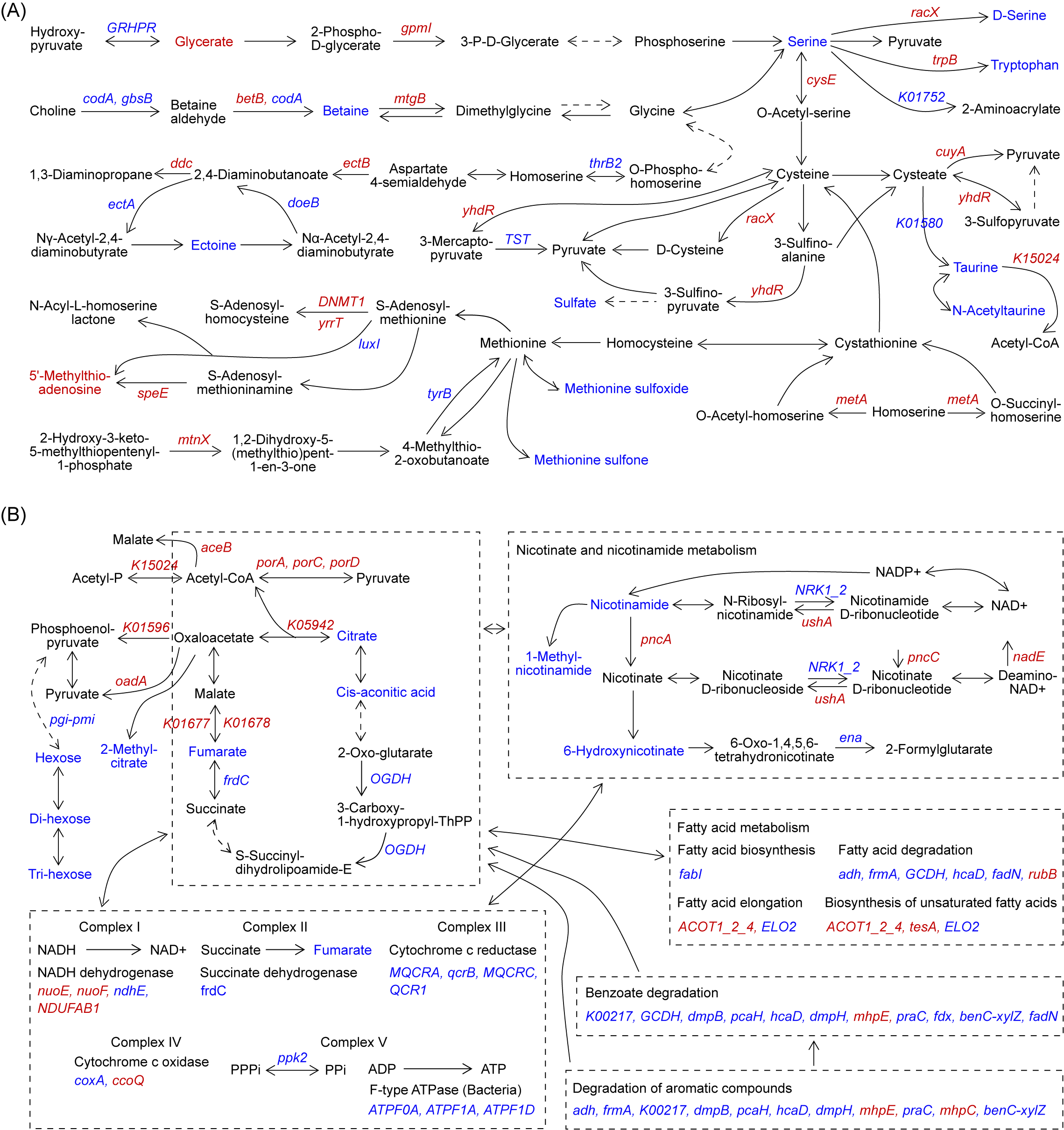


Figure S10 Polystyrene microplastic (PS) exposure induces metabolic dysfunctions of other pathways involved in redox homeostasis in gut microbiota. (A) Changes in glycine, serine and threonine metabolism, cysteine and methionine metabolism, and taurine and hypotaurine metabolism. (B) Changes in the tricarboxylic acid cycle, oxidative phosphorylation, nicotinate and nicotinamide metabolism, fatty acid metabolism, degradation of aromatic compounds, and benzoate degradation. Red/blue fonts: significantly increased/decreased upon PS exposure (*p* < 0.05, two-sided Mann-Whitney *U* test). Dashed arrows indicate that conversions between metabolites require at least two steps of biochemical reactions. Solid arrows indicate that biochemical conversions can occur directly between metabolites.


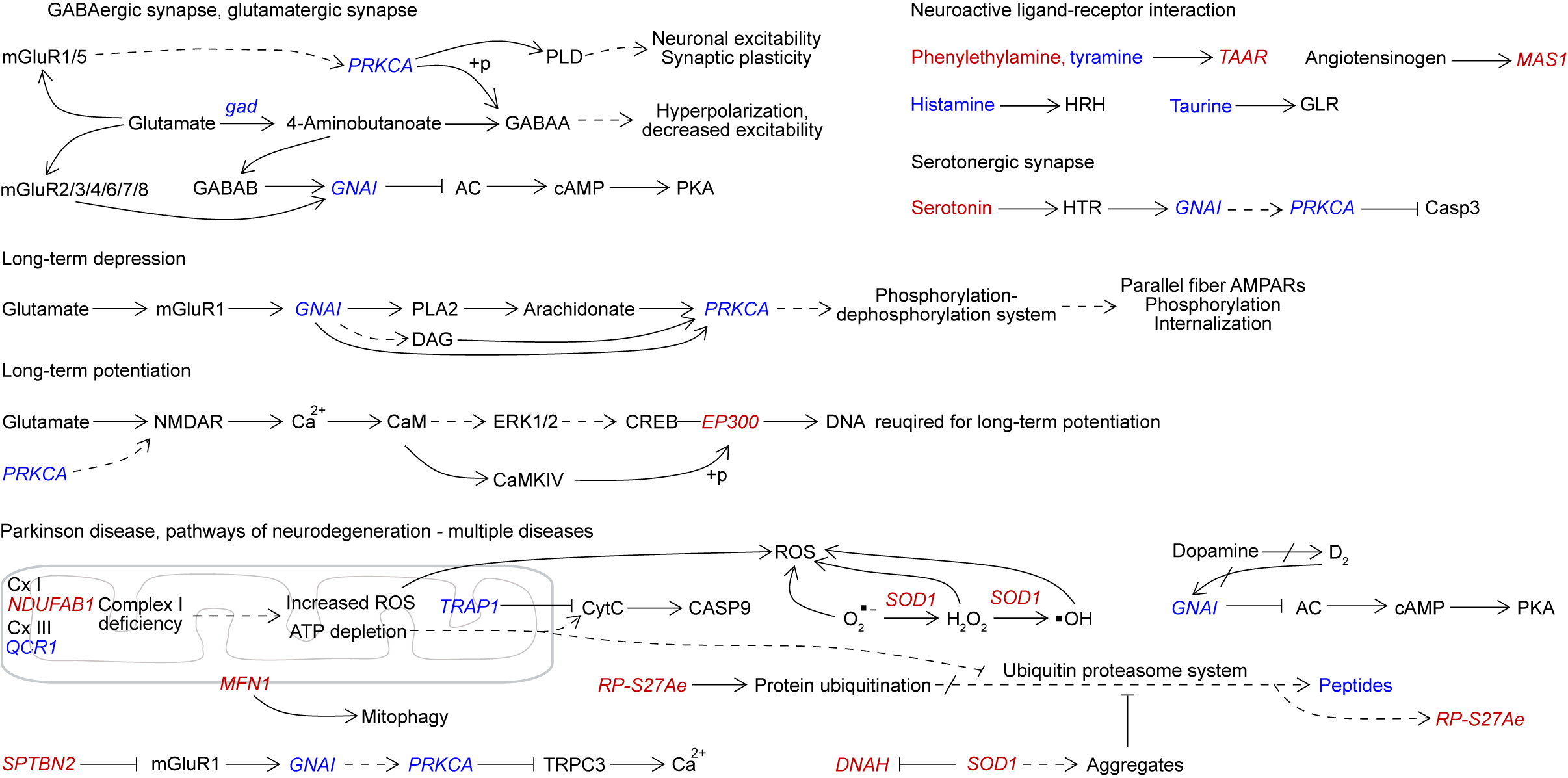


Figure S11 Polystyrene microplastic (PS) exposure induces metabolic disturbances involved in neurological functions. Red/blue fonts: significantly increased/decreased upon PS exposure (*p* < 0.05, two-sided Mann-Whitney *U* test).


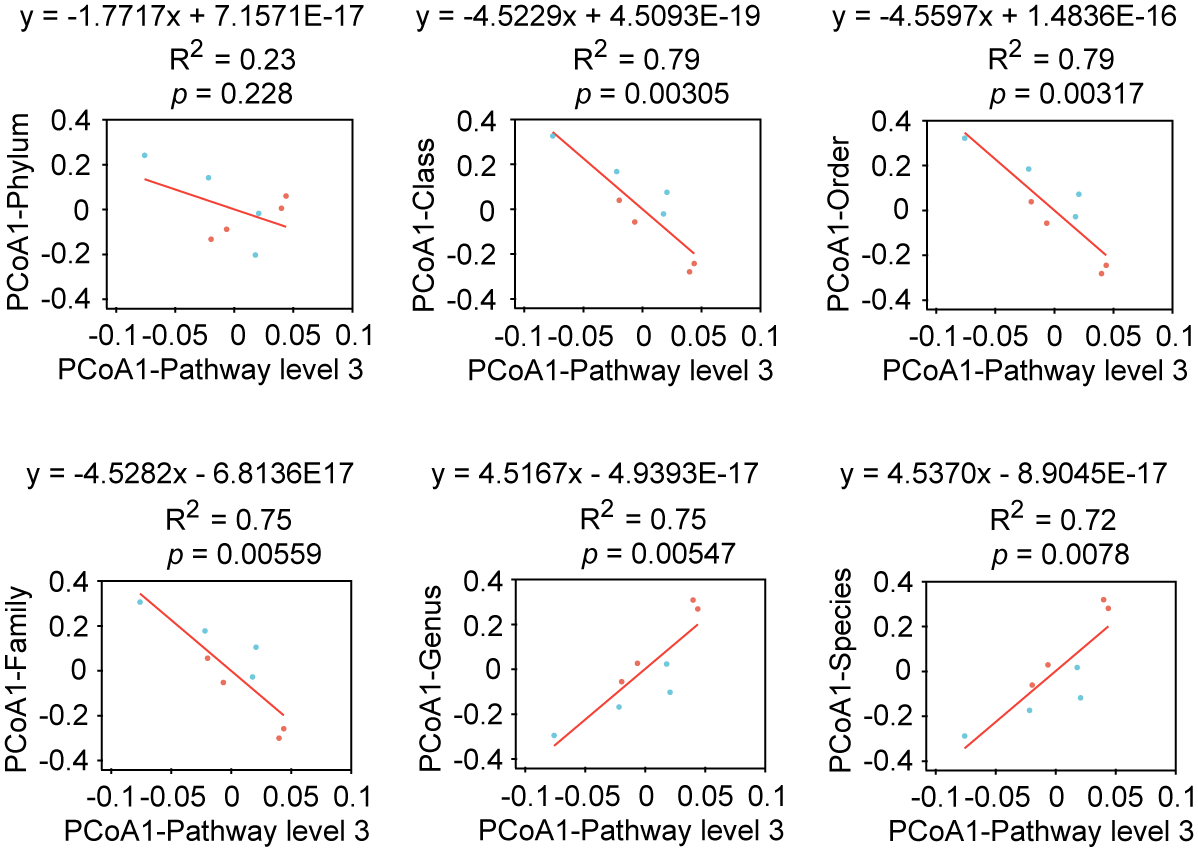


Figure S12 Regression analysis of gut microbiota and Kyoto Encyclopedia of Genes and Genomes functions from the phylum to species.


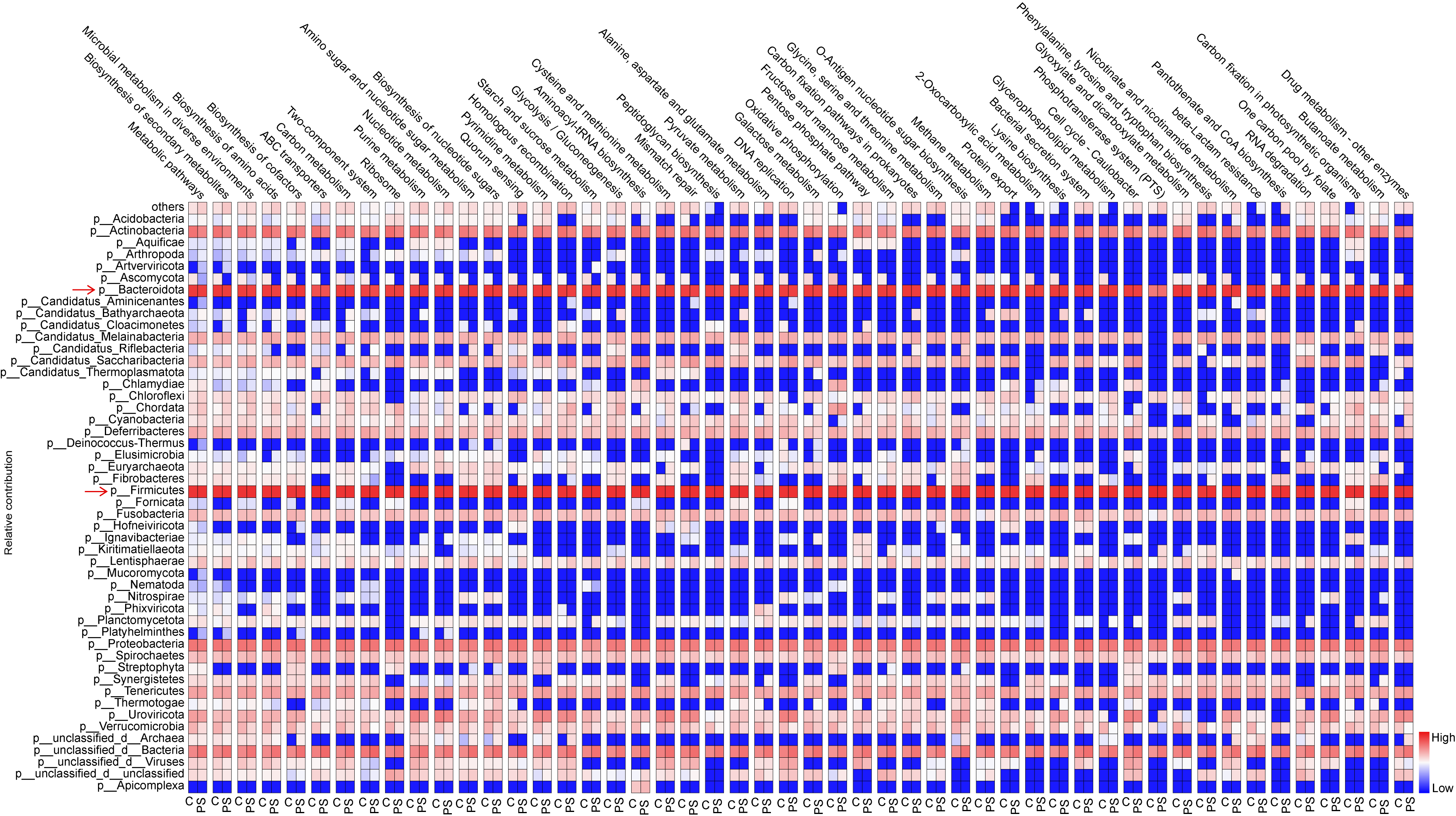


Figure S13 Species contributions of gut microbiota at the phylum level to Kyoto Encyclopedia of Genes and Genomes functions. C, the control group. PS, the polystyrene microplastic (PS) exposure group. Arrow, dominant species contributions.


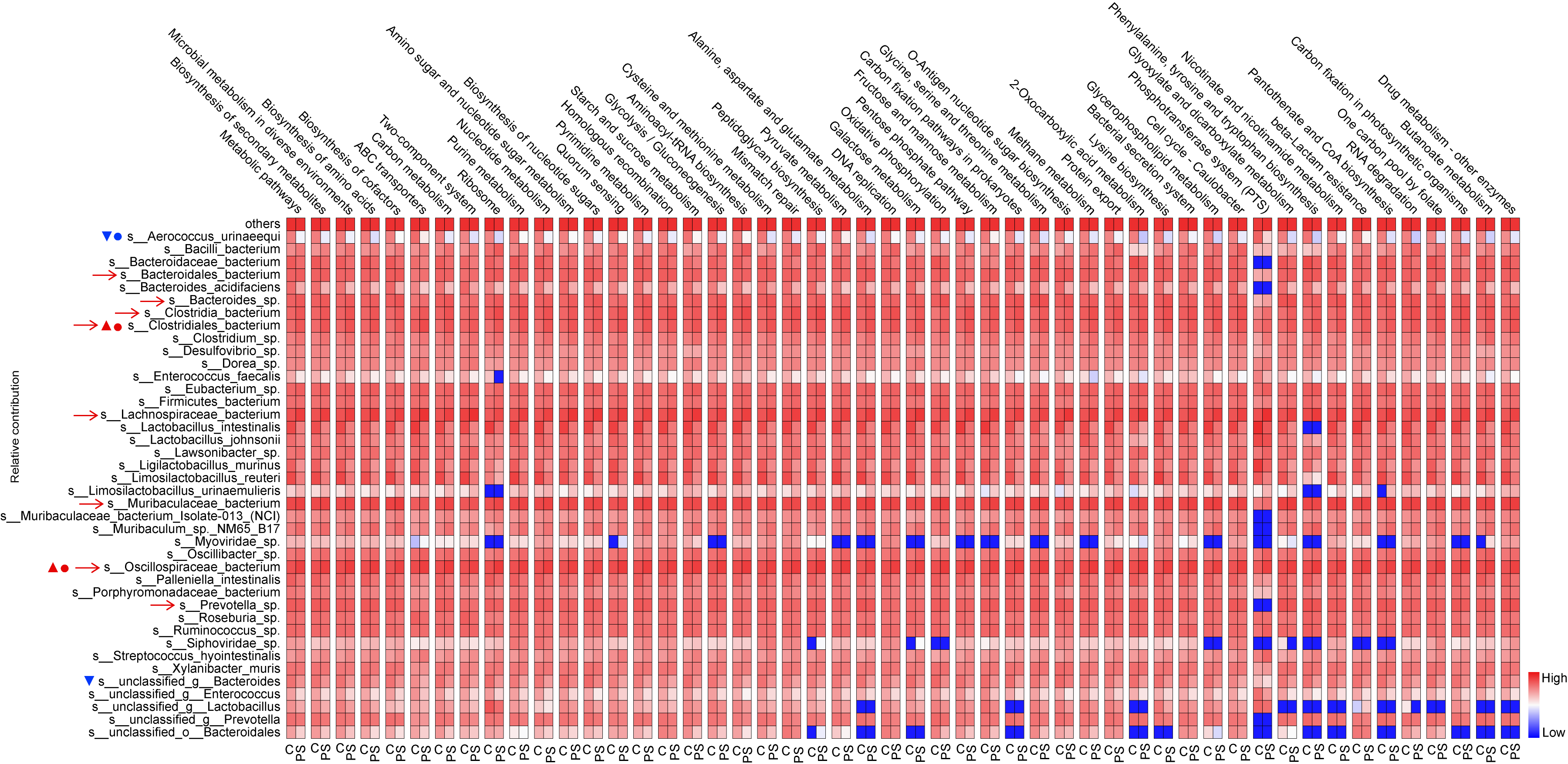


Figure S14 Species contributions of gut microbiota at the species level to Kyoto Encyclopedia of Genes and Genomes functions. C, the control group. PS, the polystyrene microplastic (PS) exposure group. Arrow, dominant species contributions. Red/blue triangle, microbial abundances significantly increased/decreased upon PS exposure (*p* < 0.05, two-sided Mann-Whitney *U* test). Circle, the top 20 most abundant microbes with significant alterations upon PS exposure (*p* < 0.05, two-sided Mann-Whitney *U* test).


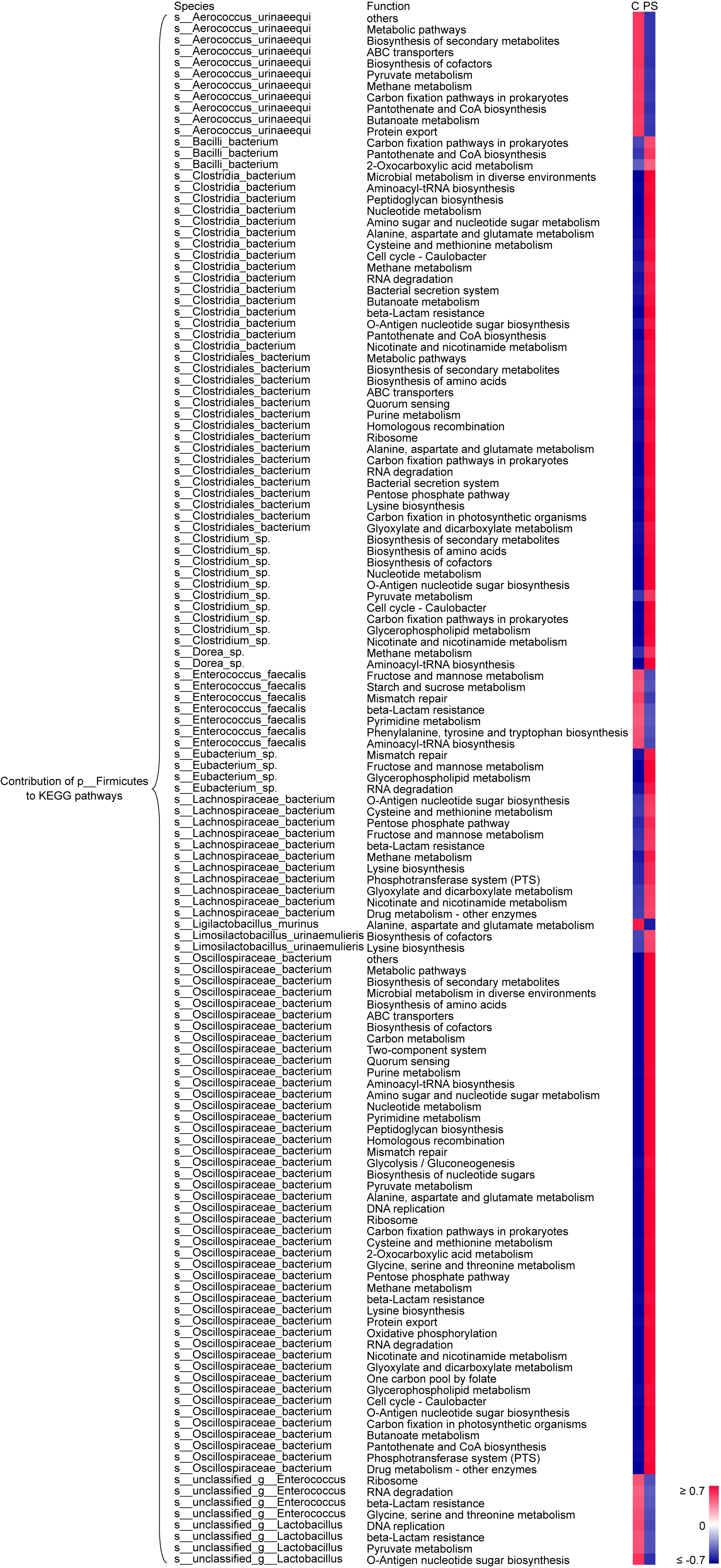


Figure S15 Polystyrene microplastic (PS) exposure induces changes in gut species contributions of the phylum Firmicutes to Kyoto Encyclopedia of Genes and Genomes (KEGG) functions at the species level. Species contributions to KEGG functions with significant alterations (*p* < 0.05, two-sided Mann-Whitney *U* test) were listed. The average of each group was used for the heat map plot.


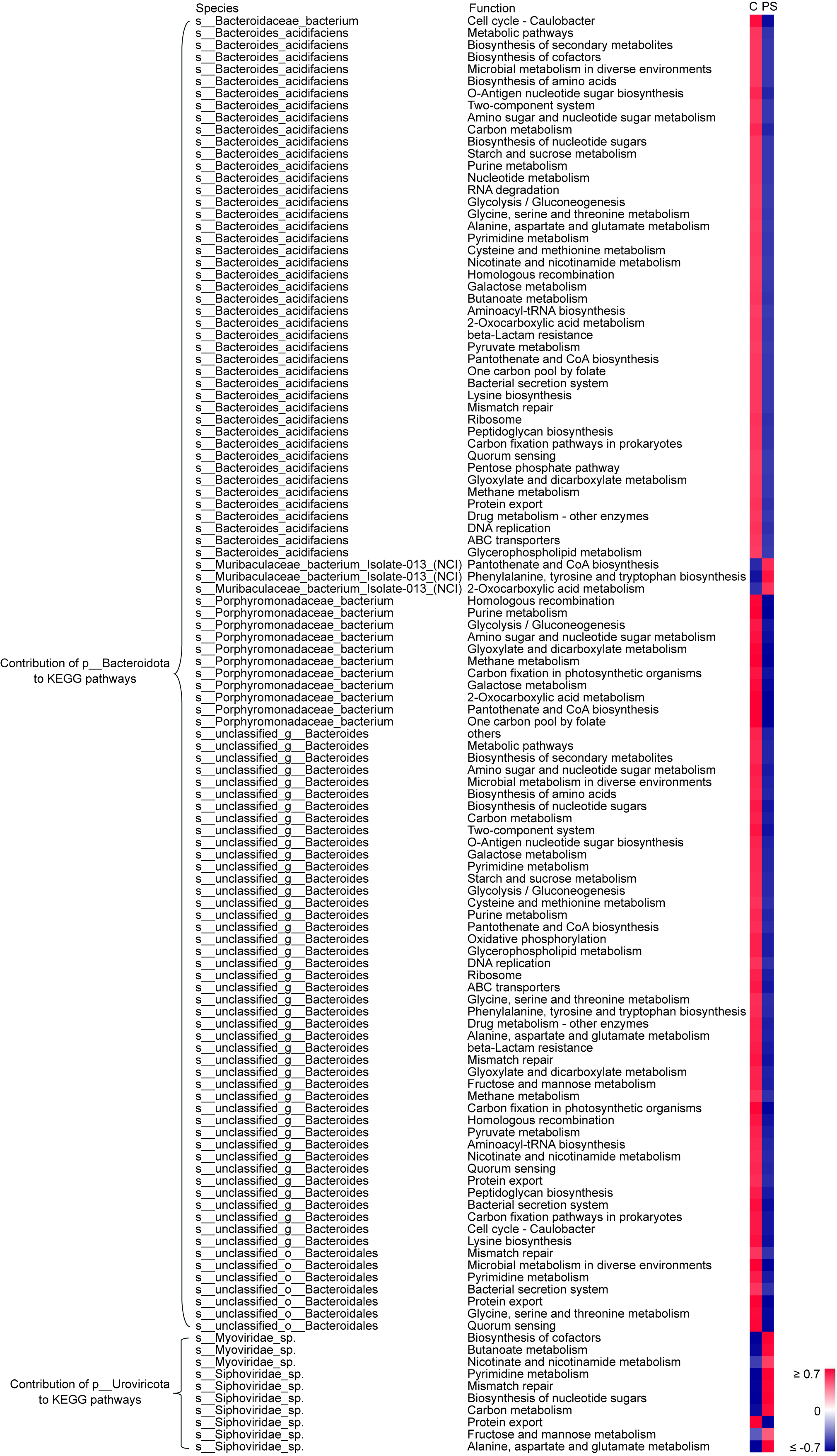


Figure S16 Polystyrene microplastic (PS) exposure induces changes in gut species contributions of the phylum Bacteroidota to Kyoto Encyclopedia of Genes and Genomes (KEGG) functions at the species level. Species contributions to KEGG functions with significant alterations (*p* < 0.05, two-sided Mann-Whitney *U* test) were listed. The average of each group was used for the heat map plot.


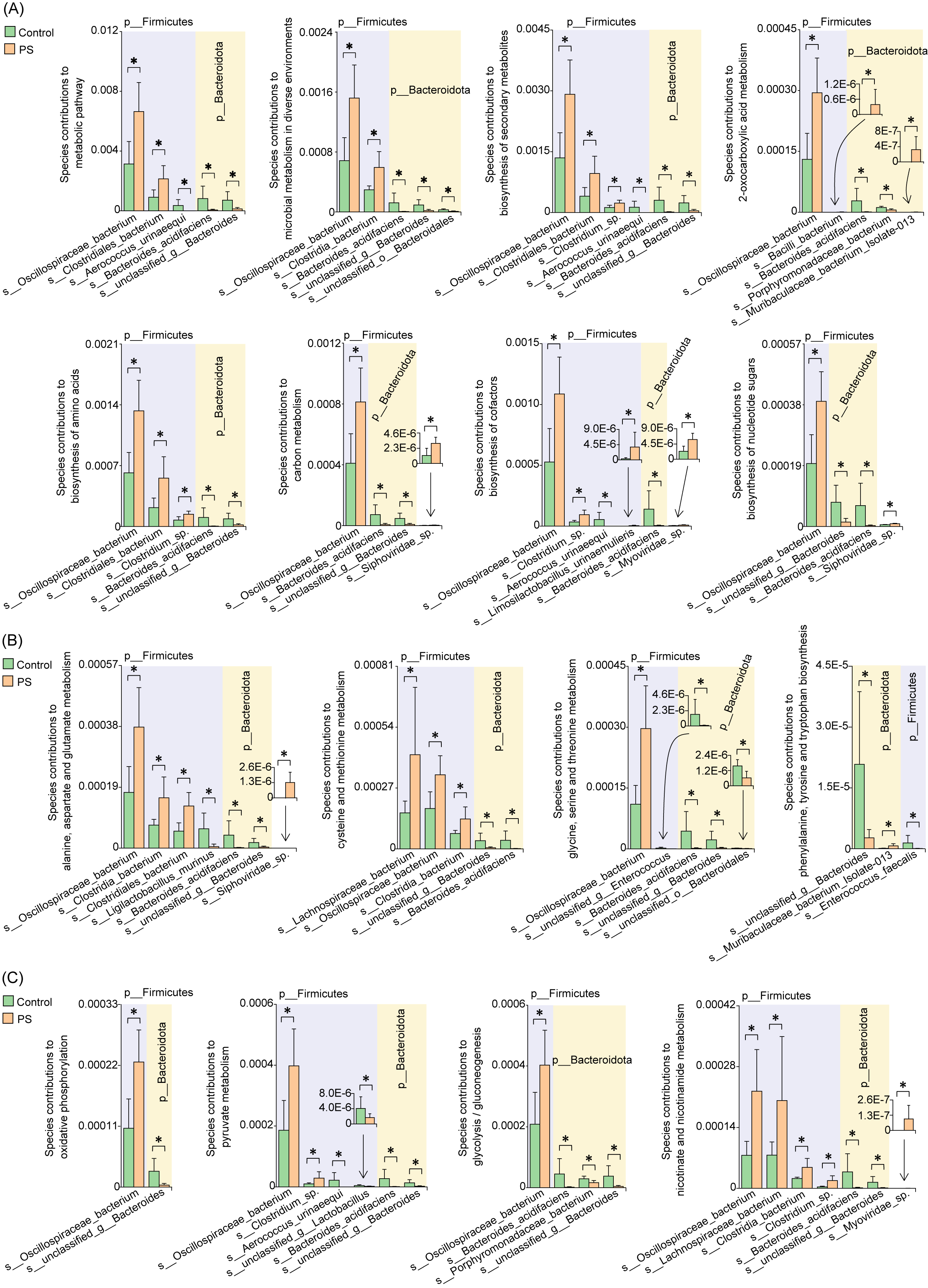


Figure S17 Changes in species contributions of gut microbiota at the species level to Kyoto Encyclopedia of Genes and Genomes functions upon PS exposure. (A) Global and overview maps of pathways. (B) Amino acid metabolism involved in glutathione and aromatic amino acids. (C) Energy metabolism. *, *p* < 0.05, two-sided Mann-Whitney *U* test. The mean plus standard deviation was used for the column plot.

**REFERENCES**

1. Carol Kilkenny, William Browne, Innes C. Cuthill, Michael Emerson, Douglas G Altman 2010. “Animal research: reporting in vivo experiments: the ARRIVE guidelines.” *British Journal Of Pharmacology* **160**: 1577−1579. https://doi.org/10.1111/j.1476-5381.2010.00872.x

2. Marfella, Raffaele, Francesco Prattichizzo, Celestino Sardu, Gianluca Fulgenzi, Laura Graciotti, Tatiana Spadoni, Nunzia D'Onofrio, et al. 2024. “Microplastics and nanoplastics in atheromas and cardiovascular events.” *New England Journal of Medicine* **390**: 900−910. https://doi.org/10.1056/NEJMoa2309822

3. Cox, Kieran D., Garth A. Covernton, Hailey L. Davies, John F. Dower, Francis Juanes, Sarah E. Dudas. 2019. “Human consumption of microplastics.” *Environmental Science & Technology* **53**: 7068−7074. https://doi.org/10.1021/acs.est.9b01517

4. Jeong, Bohyeon, Jeong Yeob Baek, Jahong Koo, Subin Park, Young-Kyoung Ryu, Kyoung-Shim Kim, Seungjae Zhang, et al. 2022. “Maternal exposure to polystyrene nanoplastics causes brain abnormalities in progeny.” *Journal Of Hazardous Materials* **426**: 127815. https://doi.org/10.1016/j.jhazmat.2021.127815

5. Pivokonsky, Martin, Lenka Cermakova, Katerina Novotna, Petra Peer, Tomas Cajthaml, Vaclav Janda. 2018. “Occurrence of microplastics in raw and treated drinking water.” *Science Of The Total Environment* **643**: 1644−1651. https://doi.org/10.1016/j.scitotenv.2018.08.102

6. Pivokonský, Martin, Lenka Pivokonská, Kateřina Novotná, Lenka Čermáková, Martina Klimtová. 2020. “Occurrence and fate of microplastics at two different drinking water treatment plants within a river catchment.” *Science Of The Total Environment* **741**: 140236. https://doi.org/10.1016/j.scitotenv.2020.140236

7. Chen, Yunqian, Jiangbin Shu, Chunlin Li, Xingnan Ye, Qing Li, Christian George, Jianmin Chen. 2025. “Size distribution of micro-/nanoplastic particles and their chemical speciation in the atmosphere of Shanghai, China.” *Environmental Science & Technology*. https://doi.org/10.1021/acs.est.5c03278

8. Nihart, Alexander J., Marcus A. Garcia, Eliane El Hayek, Rui Liu, Marian Olewine, Josiah D. Kingston, Eliseo F. Castillo, et al. 2025. “Bioaccumulation of microplastics in decedent human brains.” *Nature Medicine* **31**: 1114−1119. https://doi.org/10.1038/s41591-024-03453-1

9. Amato-Lourenço, Luís Fernando, Regiani Carvalho-Oliveira, Gabriel Ribeiro Júnior, Luciana Dos Santos Galvão, Rômulo Augusto Ando, Thais Mauad. 2021. “Presence of airborne microplastics in human lung tissue.” *Journal Of Hazardous Materials* **416**: 126124. https://doi.org/10.1016/j.jhazmat.2021.126124

10. Montano, Luigi, Elisabetta Giorgini, Valentina Notarstefano, Tiziana Notari, Maria Ricciardi, Marina Piscopo, Oriana Motta. 2023. “Raman Microspectroscopy evidence of microplastics in human semen.” *Science Of The Total Environment* **901**: 165922. https://doi.org/10.1016/j.scitotenv.2023.165922

11. Zhang, Lin, Jiaqi Tian, Xiaodan Zhu, Linlin Wang, Xiang Yun, Liyang Liang, Shuyin Duan. 2025. “Cross-platform detection of microplastics in human biological tissues: Comparing spectroscopic and chromatographic approaches.” *Journal Of Hazardous Materials* **492**: 138133. https://doi.org/10.1016/j.jhazmat.2025.138133

12. Tian, Jiaqi, Liyang Liang, Qiang Li, Ning Li, Xiaodan Zhu, Lin Zhang. 2025. “Association between microplastics in human amniotic fluid and pregnancy outcomes: Detection and characterization using Raman spectroscopy and pyrolysis GC/MS.” *Journal Of Hazardous Materials* **482**: 136637. https://doi.org/10.1016/j.jhazmat.2024.136637

13. Yun, Xiang, Liyang Liang, Jiaqi Tian, Ning Li, Zhen Chen, Yongfei Zheng, Shuyin Duan, Lin Zhang. 2024. “Raman-guided exploration of placental microplastic exposure: Unraveling the polymeric tapestry and assessing developmental implications.” *Journal Of Hazardous Materials* **477**: 135271. https://doi.org/10.1016/j.jhazmat.2024.135271

14. Weingrill, Rodrigo Barbano, Men-Jean Lee, Paula Benny, Jonathan Riel, Kevin Saiki, Jacob Garcia, Lais Farias Azevedo de Magalhaes Oliveira, et al. 2023. “Temporal trends in microplastic accumulation in placentas from pregnancies in Hawai'i.” *Environment International* **180**: 108220. https://doi.org/10.1016/j.envint.2023.108220

15. Ye, Guozhu, Xu Zhang, Changzhou Yan, Yi Lin, Qiansheng Huang. 2021. “Polystyrene microplastics induce microbial dysbiosis and dysfunction in surrounding seawater.” *Environment International* **156**: 106724. https://doi.org/https://doi.org/10.1016/j.envint.2021.106724

16. Zhang, Xu, Kai Wen, Dongxiao Ding, Jintao Liu, Zhao Lei, Xiaoxuan Chen, Guozhu Ye, et al. 2021. “Size-dependent adverse effects of microplastics on intestinal microbiota and metabolic homeostasis in the marine medaka (Oryzias melastigma).” *Environment International* **151**: 106452. https://doi.org/10.1016/j.envint.2021.106452

17. Ye, Guozhu, Xu Zhang, Xinyu Liu, Xu Liao, Han Zhang, Changzhou Yan, Yi Lin, Qiansheng Huang. 2021. “Polystyrene microplastics induce metabolic disturbances in marine medaka (Oryzias melastigmas) liver.” *Science Of The Total Environment* **782**: 146885. https://doi.org/10.1016/j.scitotenv.2021.146885

18. Zhang, Zhu, Wenqing Chen, Hiutung Chan, Junjie Peng, Peili Zhu, Junkui Li, Xiaoli Jiang, et al. 2024. “Polystyrene microplastics induce size-dependent multi-organ damage in mice: Insights into gut microbiota and fecal metabolites.” *Journal Of Hazardous Materials* **461**: 132503. https://doi.org/10.1016/j.jhazmat.2023.132503

19. Chan, Wan Ting, Carl Angelo Medriano, Sungwoo Bae. 2023. “Unveiling the impact of short-term polyethylene microplastics exposure on metabolomics and gut microbiota in earthworms (Eudrilus euganiae).” *Journal Of Hazardous Materials* **460**: 132305. https://doi.org/10.1016/j.jhazmat.2023.132305

20. Jin, Haibo, Tan Ma, Xiaoxuan Sha, Zhenyu Liu, Yuan Zhou, Xiannan Meng, Yabing Chen, Xiaodong Han, Jie Ding. 2021. “Polystyrene microplastics induced male reproductive toxicity in mice.” *Journal Of Hazardous Materials* **401**: 123430. https://doi.org/10.1016/j.jhazmat.2020.123430

21. Zhang, Kaikai, Jianzheng Yang, Lijian Chen, Jietao He, Dong Qu, Zheng Zhang, Yi Liu, et al. 2023. “Gut microbiota participates in polystyrene microplastics-induced hepatic injuries by modulating the gut–liver axis.” *ACS Nano* **17**: 15125−15145. https://doi.org/10.1021/acsnano.3c04449

22. Xiang, Qian, Dong Zhu, Qing-Lin Chen, Patrick O'Connor, Xiao-Ru Yang, Min Qiao, Yong-Guan Zhu. 2019. “Adsorbed sulfamethoxazole exacerbates the effects of polystyrene (∼2 μm) on gut microbiota and the antibiotic resistome of a soil collembolan.” *Environmental Science & Technology* **53**: 12823−12834. https://doi.org/10.1021/acs.est.9b04795

23. Wen, Jing, Hang Sun, Bingwei Yang, Erqun Song, Yang Song, Guibin Jiang. 2024. “Environmentally relevant concentrations of microplastic exposure cause cholestasis and bile acid metabolism dysregulation through a gut-liver loop in mice.” *Environmental Science & Technology* **58**: 1832−1841. https://doi.org/10.1021/acs.est.3c07108

24. Chen, Shifu, Yanqing Zhou, Yaru Chen, Jia Gu. 2018. “fastp: an ultra-fast all-in-one FASTQ preprocessor.” *Bioinformatics* **34**: i884−i890. https://doi.org/10.1093/bioinformatics/bty560

25. Dinghua Li, Chi-Man Liu, Ruibang Luo, Kunihiko Sadakane, Tak-Wah Lam. 2015. “MEGAHIT: an ultra-fast single-node solution for large and complex metagenomics assembly via succinct de Bruijn graph.” *Bioinformatics* **31**: 1674−1676. https://doi.org/10.1093/bioinformatics/btv033

26. Doug Hyatt, Gwo-Liang Chen, Philip F. Locascio, Miriam L. Land, Frank W. Larimer, Loren J. Hauser. 2010. “Prodigal: prokaryotic gene recognition and translation initiation site identification.” *BMC Bioinformatics* **11**: 119. https://doi.org/10.1186/1471-2105-11-119

27. Limin Fu, Beifang Niu, Zhengwei Zhu, Sitao Wu, Weizhong Li. 2012. “CD-HIT: accelerated for clustering the next-generation sequencing data.” *Bioinformatics* **28**: 3150−3152. https://doi.org/10.1093/bioinformatics/bts565

28. Qin, Junjie, Yingrui Li, Zhiming Cai, Shenghui Li, Jianfeng Zhu, Fan Zhang, Suisha Liang, et al. 2012. “A metagenome-wide association study of gut microbiota in type 2 diabetes.” *Nature* **490**: 55−60. https://doi.org/10.1038/nature11450

29. Benjamin Buchfink, Chao Xie, Daniel H. Huson. 2015. “Fast and sensitive protein alignment using DIAMOND.” *Nature Methods* **12**: 59−60. https://doi.org/10.1038/nmeth.3176

30. Chen Xie, Xizeng Mao, Jiaju Huang, Yang Ding, Jianmin Wu, Shan Dong, Lei Kong, Ge Gao, Chuan-Yun Li, Liping Wei. 2011. “KOBAS 2.0: a web server for annotation and identification of enriched pathways and diseases.” *Nucleic Acids Research* **39**: W316−W322. https://doi.org/10.1093/nar/gkr483

31. Ye, Guozhu, Dongxiao Ding, Han Gao, Yulang Chi, Jinsheng Chen, Zeming Wu, Yi Lin, Sijun Dong. 2019. “Comprehensive metabolic responses of HepG2 cells to fine particulate matter exposure: Insights from an untargeted metabolomics.” *Science Of The Total Environment* **691**: 874−884. https://doi.org/https://doi.org/10.1016/j.scitotenv.2019.07.192

32. Wishart, David S., AnChi Guo, Eponine Oler, Fei Wang, Afia Anjum, Harrison Peters, Raynard Dizon, et al. 2022. “HMDB 5.0: the human metabolome database for 2022.” *Nucleic Acids Res* **50**: D622−D31. https://doi.org/10.1093/nar/gkab1062

33. Sumner, Lloyd W., Alexander Amberg, Dave Barrett, Michael H. Beale, Richard Beger, Clare A. Daykin, Teresa W-M Fan, et al. 2007. “Proposed minimum reporting standards for chemical analysis Chemical Analysis Working Group (CAWG) Metabolomics Standards Initiative (MSI).” *Metabolomics* **3**: 211−221. https://doi.org/10.1007/s11306-007-0082-2

34. Jessica D. Ewald, Guangyan Zhou, Yao Lu, Jelena Kolic, Cara Ellis, James D. Johnson, Patrick E. Macdonald, Jianguo Xia. 2024. “Web-based multi-omics integration using the Analyst software suite.” *Nature Protocols* **19**: 1467−1497. https://doi.org/10.1038/s41596-023-00950-4

35. Alexander I Saeed, Nirmal K Bhagabati, John C. Braisted, Wei Liang, Vasily Sharov, Eleanor A. Howe, Jianwei Li, Mathangi Thiagarajan, Joseph A. White, John Quackenbush. 2006. “TM4 microarray software suite.” *Methods In Enzymology* **411**: 134−193. https://doi.org/10.1016/S0076-6879(06)11009-5
